# Two‐Step Validation Approach for Tools To Study the DNA Repair Enzyme SNM1A

**DOI:** 10.1002/cbic.202200756

**Published:** 2023-06-02

**Authors:** Ellen M. Fay, Ailish Newton, Mark Berney, Afaf H. El‐Sagheer, Tom Brown, Joanna F. McGouran

**Affiliations:** ^1^ School of Chemistry and Trinity Biomedical Sciences Institute Trinity College Dublin The University of Dublin Dublin 2 D02 R590 Ireland; ^2^ Department of Chemistry University of Oxford Mansfield Road OX1 3TA Oxford UK

**Keywords:** DNA repair, fragment-based screening, metal-binding groups, oligonucleotide modification, SNM1A

## Abstract

We report a two‐step validation approach to evaluate the suitability of metal‐binding groups for targeting DNA damage‐repair metalloenzymes using model enzyme SNM1A. A fragment‐based screening approach was first used to identify metal‐binding fragments suitable for targeting the enzyme. Effective fragments were then incorporated into oligonucleotides using the copper‐catalysed azide–alkyne cycloaddition reaction. These modified oligonucleotides were recognised by SNM1A at >1000‐fold lower concentrations than their fragment counterparts. The exonuclease SNM1A is a key enzyme involved in the repair of interstrand crosslinks, a highly cytotoxic form of DNA damage. However, SNM1A and other enzymes of this class are poorly understood, as there is a lack of tools available to facilitate their study. Our novel approach of incorporating functional fragments into oligonucleotides is broadly applicable to generating modified oligonucleotide structures with high affinity for DNA damage‐repair enzymes.

## Graphical abstract

**A two‐step validation approach** can evaluate metal‐binding groups that are suitable for targeting the active site of the DNA damage‐repair enzyme SNM1A. A fragment‐based screening approach first identified metal‐binding fragments suitable for targeting the enzyme. Effective fragments were then incorporated into oligonucleotides using a CuAAC reaction; the modified oligonucleotides showed effective recognition by SNM1A.

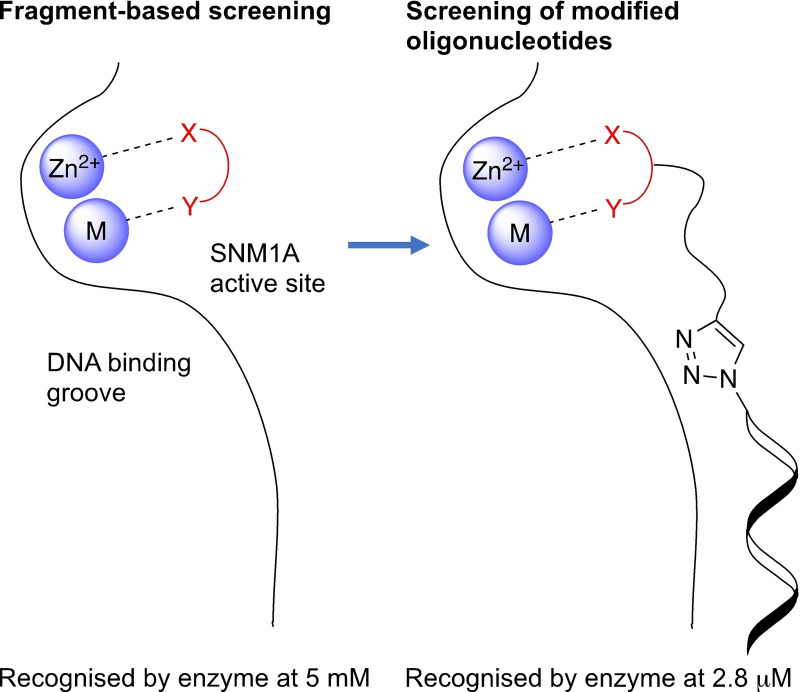

## Introduction

Metallo‐β‐lactamases (MBLs) are an important enzyme superfamily present in all domains of life. The characteristic structural motif of MBLs is a fold domain enabling transition metal‐binding which is essential for enzyme activity.^[1]^ In humans, MBLs perform a wide variety of functions including amide, ester, thioester and phosphodiester hydrolysis.^[1]^ Nucleases SNM1A and SNM1B, members of this enzyme class, are key in the repair of DNA interstrand crosslinks, highly cytotoxic forms of DNA damage.^[2]^ Cells depleted in SNM1A show increased sensitivity to certain chemotherapeutics such as cisplatin,^[2b]^ and therefore SNM1A demonstrates potential as a novel target for treating chemotherapy resistant cancers.

**Figure 1:**
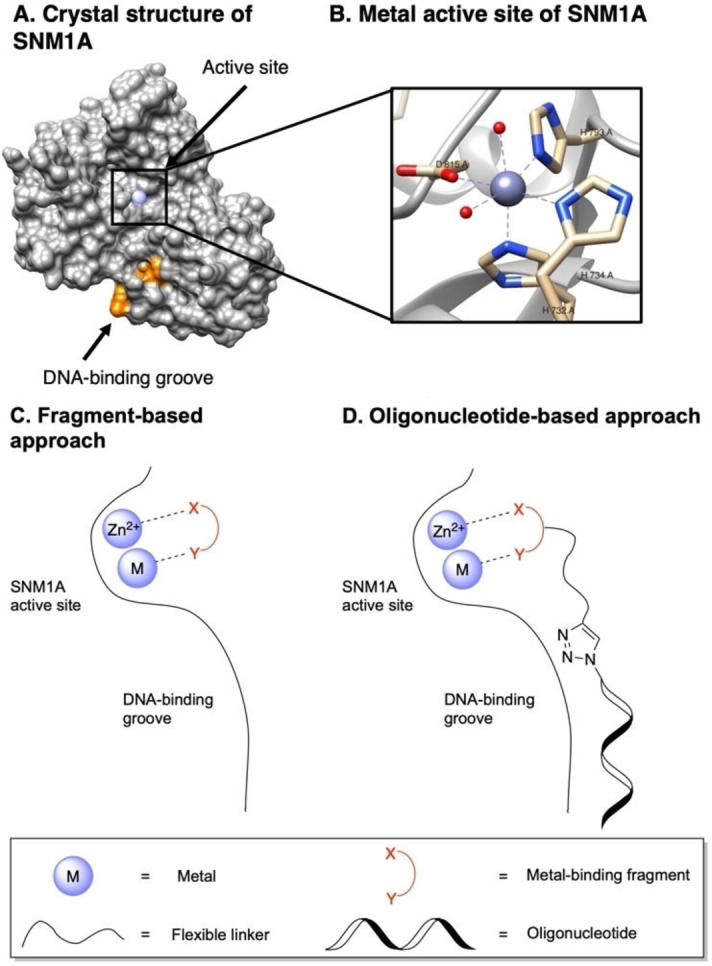
Two‐step approval method for the rapid identification of metal‐binding groups suitable for targeting SNM1A. A) Crystal structure of SNM1A (PDB ID: 5AHR) showing the metal active site (purple) and DNA‐binding groove (orange). Graphic created using UCSF Chimera.^[13]^ B) Active site of SNM1A showing coordination of a Zn^II^ ion. C) Fragment‐based approach for targeting the active site. D) Oligonucleotide‐based approach for targeting the active site and DNA‐binding groove of SNM1A. Metal‐binding fragments were incorporated into oligonucleotide strands using the CuAAC reaction. Modified oligonucleotides were then evaluated for recognition by SNM1A.

SNM1A is a 5’‐exonuclease that hydrolyses the phosphodiester backbone of DNA.^[3]^ SNM1A is capable of digesting past various DNA lesions,^[4]^ however, a phosphate group is required on the 5’‐position of substrate DNA.^[3]^ A crystal structure of truncated SNM1A obtained by Allerston et al. contains a single Zn^II^ ion in the active site (Figure 1A, B).^[5]^ However, it is postulated that the active form of SNM1A accommodates a second metal cation as in the crystal structure of SNM1B.[^4^, ^5^] It is thought that this metal active site is key for priming the phosphodiester bond for hydrolysis. SNM1A also contains a phosphate‐binding pocket,^[3]^ to facilitate phosphodiester hydrolysis. Furthermore, the crystal structure of SNM1A reveals the presence of a groove with the dimensions appropriate for the accommodation of DNA strands (Figure 1A).^[5]^ Mutational analysis identified lysine residues in this distal DNA binding region that are required for efficient digestion of DNA.^[5]^ Hence, the interaction of the enzyme and the DNA strand is key for enzyme activity.

Due to these substrate recognition requirements, few inhibitors of SNM1A have been identified to date. The β‐lactam antibiotics cephalosporins, prototypical MBL inhibitors, show strong SNM1A inhibition.^[6]^ Incorporation of metal‐binding groups into phosphorylated nucleoside derivatives is an effective method of generating inhibitors of SNM1A.^[7]^ The incorporation of a nucleoside/oligonucleotide scaffold has proven to enhance substrate recognition and increase binding affinity.^[8]^ However, the synthesis of native nucleosides and oligonucleotides to evaluate the recognition of novel substrates is challenging, time‐consuming and expensive.^[9]^ Synthesis of modified nucleosides suitable for solid phase oligonucleotide synthesis typically includes upwards of six synthetic steps prior to generation of the phosphoramidites which are synthetically difficult to handle due to their instability towards H_2_O and O_2_.^[8]^ New, facile synthetic and screening methodologies are therefore required to rapidly evaluate the efficiency of substrate binding and to access non‐native SNM1A substrates. Although screens of bioactive molecules have previously succeeded in identifying several inhibitors of SNM1A,[^6^, ^10^] there is currently no screening method focused solely on the identification of metal‐binding groups, which have been demonstrated as being key for targeting SNM1A.[^7^, ^8^, ^11^]

Fragment‐based screening approaches evaluate the ability of low‐molecular‐weight molecules to exert a desired biological action.^[12]^ Although the fragments themselves may only have low binding affinity for the desired target, they can be combined with other scaffolds or functional groups to create a final drug molecule or tool compound with high binding affinity. As fragments often have low affinity for the biological target, they are usually screened at high concentrations.[^12^, ^14^] Several drugs derived from fragment‐based screening have been approved by the FDA, including vemurafenib, used in the treatment of late‐stage melanoma.^[15]^ Fragment‐based screening has been extensively used as an initial screening method and has been successful in generating new hits for inhibitors of both DNA processing enzymes, such as the DNA damage repair enzyme MRE11,^[16]^ and metalloenzymes, including the metallo‐β‐lactamase NDM‐1.^[17]^ However, fragment‐based screening has not yet been used to identify inhibitors of SNM1A.

The use of click chemistry and the copper catalysed azide–alkyne cycloaddition (CuAAC) reaction to modify DNA has been extensively studied and reviewed.^[18]^ Modifications installed using click chemistry include fluorophores,^[19]^ affinity handles^[20]^ and metal complexes.^[21]^ Over the last few years, there has been vast research into the generation of biocompatible oligonucleotides with a triazole linkage replacing the phosphodiester bond.^[22]^ Triazole‐modified oligonucleotides can be replicated and transcribed in cells.^[23]^ Incorporation of fragments into peptides by click chemistry to explore protein–protein interactions has been reported.^[24]^ However, incorporation of fragments into DNA to examine DNA‐protein interactions has yet to be explored. DNA‐encoded fragment libraries have been used for identification of small molecule ligands of protein targets.^[25]^ However, in this area of research, the DNA strand is important for the construction and identification of the fragment library.^[25]^ In this work, the oligonucleotide strand is key for recognition by SNM1A.

We describe a facile two‐step approval method for the rapid identification of metal binding fragments suitable for targeting SNM1A. First, we used a fragment‐based approach (Figure 1C) to identify metal binding fragments that target the enzyme. Then, we confirmed the potency of these metal binding fragments by incorporating them into oligonucleotide scaffolds using click chemistry (Figure 1D). Whereas the fragments were recognised by SNM1A at a concentration of 5 mM, these modified oligonucleotides were recognised by SNM1A at the >1000‐fold lower concentration of 2.8 μM.

## Results and Discussion

### Fragment‐based screening for identifying metal binding fragments suitable for targeting SNM1A

**Figure 2:**
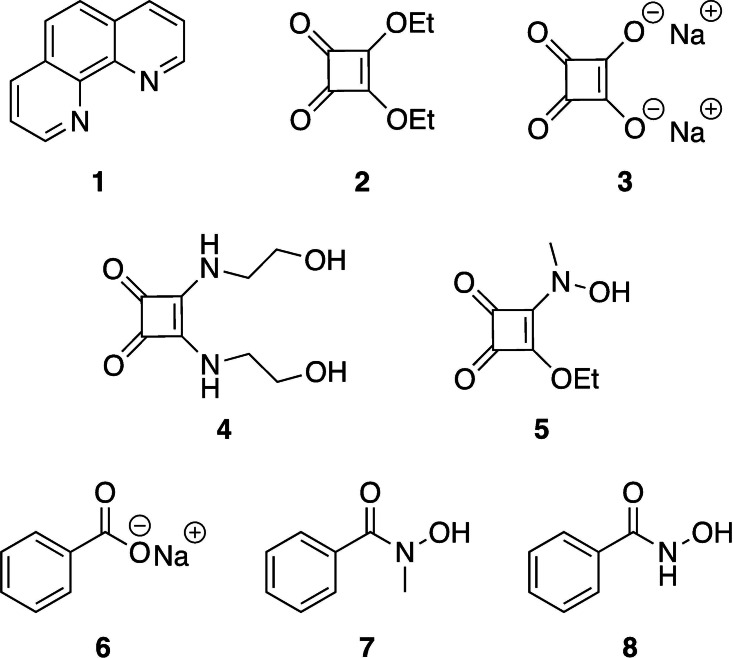
Fragments chosen for fragment‐based screening approach. Fragments were selected based on their reported metal‐binding abilities.

**Scheme 1:**
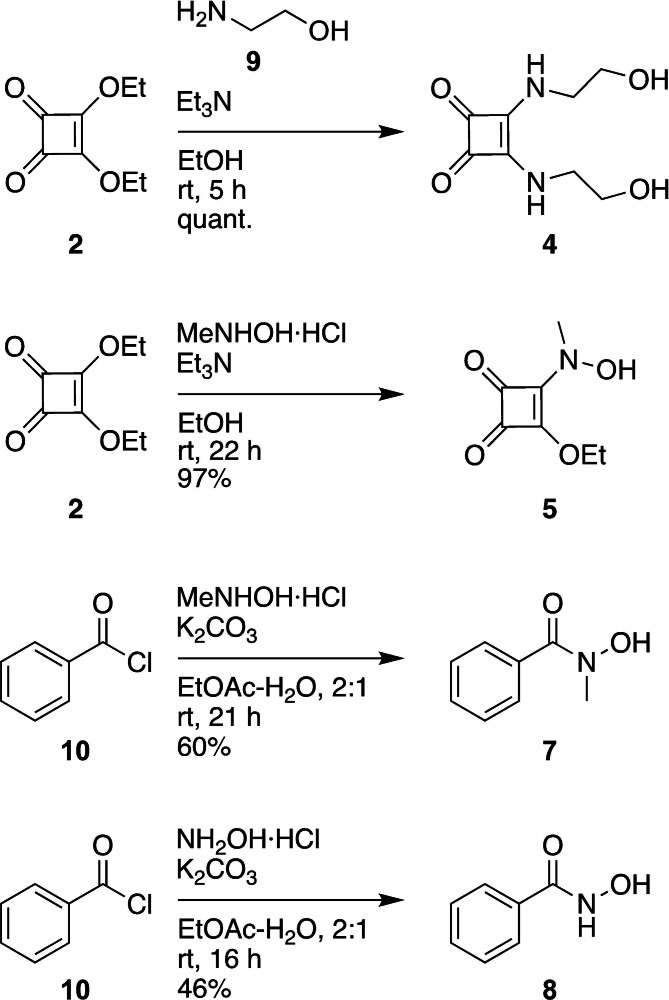
Synthesis of fragments **4**, **5**, **7** and **8**. All other fragments were commercially available.

Herein, we explore a fragment‐based screening approach for the identification of metal binding fragments suitable for targeting SNM1A (Figure 1C). Successful implementation of this method would allow a wide array of metal binding fragments to be rapidly screened for inhibition of DNA damage repair enzymes prior to their incorporation into nucleoside and oligonucleotide scaffolds to create tool molecules. Such a screening method could also increase the efficiency of developing inhibitors of SNM1A, leading to new insights into the binding mode and inhibition of the enzyme. Fragments were selected for inclusion in this proof‐of‐principle assay based on their known metal binding abilities^[26]^ and their previous success in targeting the active site of SNM1A when incorporated into a nucleoside or oligonucleotide scaffold. Fragments chosen for screening included squaric acid and squaramide derivatives[^7^, ^8^, ^11a^] as well as benzoic acid and hydroxamic acid derivatives (Figure 2).[^11b^, ^11c^] *o*‐Phenanthroline **1** was included in the screening as a positive control as it is a known zinc chelator and inhibitor of SNM1A.^[5]^ All fragments were obtained commercially or in moderate to high yields in one step from commercially available starting materials. Diethyl squarate **2** is commercially available. Disodium squarate **3** and sodium benzoate **6** were generated by ion exchange from their corresponding commercially available acids. Squaramides **4** and **5** were readily synthesised from diethyl squarate **2** (Scheme 1). Hydroxamic acid derivatives **7** and **8** were synthesised from benzoyl chloride **10** (Scheme 1).

### Biological evaluation of fragments for recognition by SNM1A

**Figure 3:**
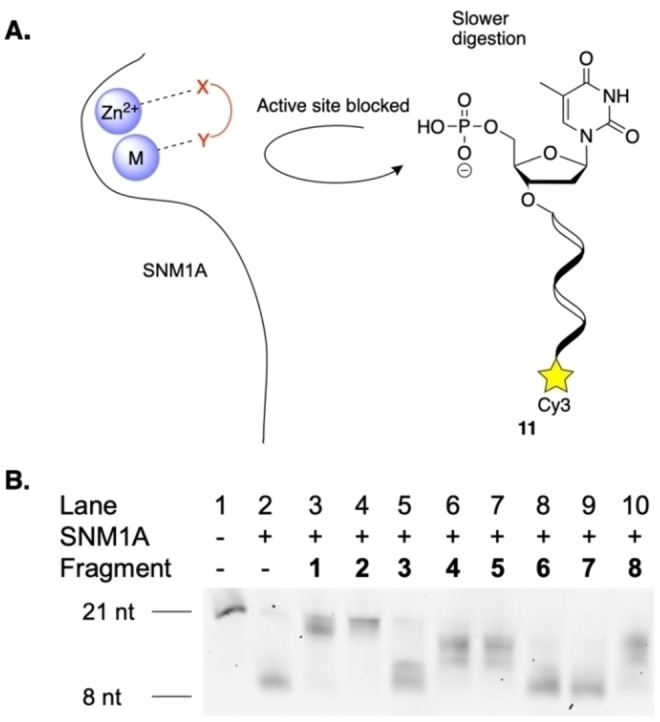
Evaluation of recognition of fragments **1**–**8** by SNM1A. A) If fragments bind to the metal centre, the active site is blocked, and fluorescent substrate **11** is not digested by the enzyme. B) Digestion of fluorescent substrate **11** (0.8 pmol) after incubation with SNM1A (50 fmol) for 60 min at 37 °C following 5 min of preincubation with fragments **1**–**8** (5 mM). Duplicate data are available in Figure S1 in the Supporting Information. nt=nucleotides; sequence of fluorescent substrate oligonucleotide **11**: 5’‐XTAG CAG TCA GTC AGT CAT CGY‐3’, X=thymidine 5’‐phosphate, Y=Cy3

Fragments **1**–**8** were evaluated for recognition by SNM1A using a gel electrophoresis‐based assay (Figure 3A).[^6^, ^8^] This assay uses 21mer oligonucleotide **11**, containing a 5’‐phosphate and a 3’‐Cy3 as a substrate for SNM1A. The 3’‐Cy3 fluorophore allows for visualisation of reaction products. As SNM1A does not show significant nuclease activity on DNA strands of eight nucleotides or fewer,^[3]^ digestion of oligonucleotide **11** to this length indicates full activity of the enzyme in this assay (Figure 3B, lane 2). The fragments were incubated at a concentration of 5 mM in solution with SNM1A at 37 °C for 5 minutes. During fragment‐based screening, fragments are often tested at high concentrations.[^12^, ^14^] 5 mM was chosen, as this is the concentration at which positive control *o*‐phenanthroline **1** has been shown to inhibit SNM1A.^[5]^ Oligonucleotide **11** was then added. Digestion of the oligonucleotide substrate **11** was examined by polyacrylamide gel electrophoresis (PAGE). In cases where hydrolysis of oligonucleotide **11** was less efficient, it is likely that the fragment has bound to the metal active site of the enzyme, inhibiting its activity. As expected, known inhibitor of SNM1A *o*‐phenanthroline **1** is recognised by SNM1A and hydrolysis of oligonucleotide **11** is hindered (Figure 3B, lane 3). Interestingly, diethyl squarate **2** (Figure 3B, lane 4) displays effective recognition by SNM1A but the disodium salt **3** (Figure 3B, lane 5) does not.

Previous studies have found that when incorporated into a nucleoside scaffold, the diethyl squarate derivative was not recognised by SNM1A whereas the sodium salt derivative was.^[11a]^ Squaric ester derivatives have been shown to react with lysine under physiological conditions.^[27]^ It is therefore possible that the electrophilic nature of **2** is causing it to act as a pan‐assay interference compound in this case and it is non‐specifically disrupting the enzyme, rather than necessarily binding to the active site. Squaramides **4** and **5** (Figure 3B, lanes 6 and 7) were moderately recognised by SNM1A. This corresponds to previous studies that showed that these metal‐binding fragments, when incorporated into a nucleoside scaffold, can inhibit SNM1A.^[11a]^ Of the benzoic acid derivatives, sodium benzoate **6** (Figure 3B, lane 8) and methylated hydroxamic acid derivative **7** (Figure 3B, lane 9), do not show any recognition by the enzyme. However, hydroxamic acid **8** (Figure 3B, lane 10) was recognised by the enzyme, hindering hydrolysis of oligonucleotide **11**. Once again, this corresponds to previous studies where a nucleoside containing a 5’‐hydroxamic acid was shown to inhibit SNM1A.^[11c]^ Fragments identified during this proof‐of‐concept assay correspond to metal‐binding groups that have previously been recognised by SNM1A.[^7^, ^8^, ^11^] This demonstrates that fragment‐based screening is a suitable approach for the identification of metal‐binding groups suitable for targeting this enzyme.

### Synthesis and screening of modified oligonucleotides

**Scheme 2:**
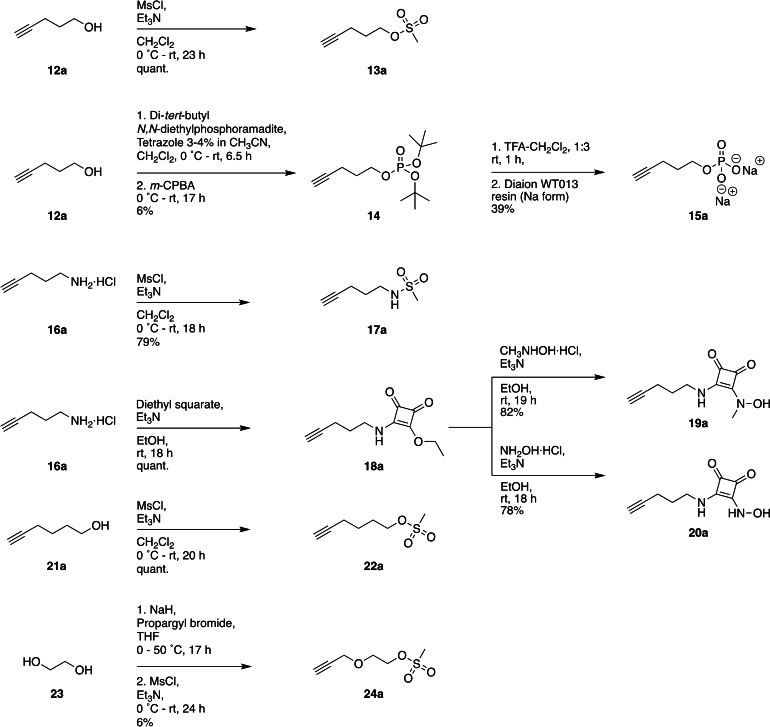
Synthesis of compounds **13 a**, **15 a**, **17 a**–**20 a**, **22 a** and **24 a** suitable for reaction with oligonucleotides using click chemistry. The compounds contain an alkyne handle, a flexible linker and a metal‐binding group.

As oligonucleotides containing a metal‐binding fragment at the 5’‐end have been shown to bind SNM1A at low concentrations,^[8]^ we hypothesised that combining the fragments with an oligonucleotide strand would allow for increased recognition by the enzyme. Inclusion of the oligonucleotide strand could enable the targeting of the distal DNA binding region of SNM1A. Oligonucleotides modified with a metal binding fragment in the 5’‐position should have a higher affinity for SNM1A than the fragments alone. DNA modification using click chemistry provided an elegant method of rapidly installing metal‐binding fragments into the 5’‐position of oligonucleotides. These modified oligonucleotides were then tested for recognition by SNM1A. We describe the synthesis of compounds containing an alkyne handle, a metal‐binding fragment, and a linker (Scheme 2). These compounds were incorporated into oligonucleotides using the CuAAC reaction, and the resulting modified oligonucleotides were evaluated for recognition by SNM1A.

A series of compounds containing an alkyne handle, a metal‐binding fragment and a flexible linker were designed. The metal‐binding fragments included the squaramide motif identified by fragment‐based screening, as well as sulfonamide groups which have previously successfully inhibited SNM1A when incorporated into a nucleoside scaffold.^[7]^ Mesylate groups were also incorporated into the oligonucleotide strand. Alcohol, amine and phosphate groups were used in this method as negative and positive binding controls. The selected triazole modification was derived from a 5’‐azido‐containing oligonucleotide and an alkyne‐containing flexible linker. This modification was selected based on the facile synthesis of the 5’‐azido oligonucleotide **25**. DNA with triazole modifications of this type can be replicated[^23b^, ^23c^] or transcribed[^23a^, ^23c^] by polymerases in cells while retaining high fidelity readthrough.[^22g^, ^23^] However, this modification displays a reduction in binding affinity to a complementary DNA or RNA target[^22f^, ^28^] and induces a slowdown in PCR replications compared to other triazole modifications.^[23c]^ This modification distorts the DNA strand when incorporated between two furanose rings.

The flexible linker was selected to allow facile coordination of the metal‐binding fragment to the active site of SNM1A by minimising perturbances caused by the triazole linkage. The linker length was also varied with a one atom extension to investigate whether the distance between the metal‐binding fragment and the oligonucleotide strand affects enzyme recognition of the modified oligonucleotide.

**Figure 4:**
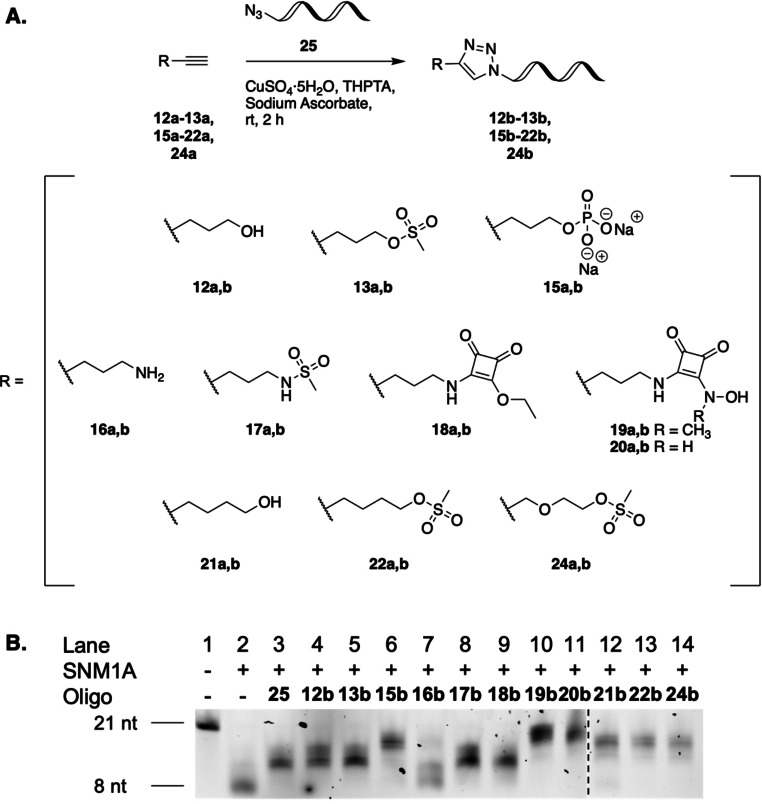
A) Late‐stage modification of oligonucleotides. Alkyne‐containing compounds **12 a**–**13 a**, **15 a**–**22 a** and **24 a** were reacted with 5’‐azido oligonucleotide **25** in a CuAAC reaction to give modified oligonucleotides **12 b**–**13 b**, **15 b**–**22 b** and **24 b**. B) Evaluation of recognition of modified oligonucleotides **12 b**–**13 b**, **15 b**–**22 b** and **24 b** by SNM1A. Digestion of fluorescent substrate **11** (0.8 pmol) after incubation with SNM1A (50 fmol) for 60 min at 37 °C following 5 min of preincubation with oligonucleotides **12 b**–**13 b**, **15 b**–**22 b** and **24 b** (2.8 μM). Duplicate data are available in Figure S*2*. nt=nucleotides; sequence of 5’‐azido oligonucleotide **25**: 5′‐(azideT)AG CAG TCA GTC AGT CAT GC‐3′; sequence of fluorescent substrate oligonucleotide **11**: 5’‐XTAG CAG TCA GTC AGT CAT CGY‐3’, X=thymidine 5’‐phosphate, Y=Cy3.

Alkyne‐containing compounds **13 a**, **15 a**, **17 a**–**20 a**, **22 a**, **24 a** were obtained in moderate to high yields in one to two steps from commercially available starting materials (Scheme 2). Mesylation of pent‐4‐yn‐1‐ol **12 a** using a literature procedure furnished mesylate **13 a** in quantitative yield.^[29]^ Pent‐4‐yn‐1‐ol **12 a** was also reacted with di‐*tert*‐butyl *N,N*‐diisopropylphosphoramidite followed by oxidation with *m*‐CPBA to give protected phosphate **14**. The phosphate group was deprotected to give the disodium phosphate **15 a**. 4‐Pent‐1‐amine hydrochloride **16 a** was mesylated to give sulfonamide **17 a** in 79 % yield. 4‐Pent‐1‐amine hydrochloride **16 a** was also reacted with diethyl squarate to furnish monosquaramide **18 a** in quantitative yield. Monosquaramide **18 a** was reacted with *N*‐methylhydroxylamine hydrochloride or hydroxylamine hydrochloride to furnish squaramides **19 a** or **20 a** in 82 % and 78 % yield, respectively. Hex‐5‐yn‐1‐ol **21 a** was also mesylated following a literature procedure to give mesylate **22 a** in quantitative yield.^[30]^ Mesylate **24 a** was generated by one‐pot propargylation‐mesylation of ethylene glycol. Compounds **12 a**–**13 a**, **15 a**–**22 a** and **24 a** were reacted with 5’‐azido oligonucleotide **25** in a CuAAC reaction to give modified oligonucleotides **12 b**–**13 b**, **15 b**–**22 b** and **24 b** (Figure 4A).

### Biological evaluation of modified oligonucleotides for recognition by SNM1A

Modified oligonucleotides **12 b**–**13 b**, **15 b**–**22 b** and **24 b** were evaluated for recognition by SNM1A in a similar biological assay to that described above. The DNA strand is highly important for substrate recognition by SNM1A.^[5]^ Therefore, whereas fragments **1**–**8** were recognised at 5 mM, modified oligonucleotides **12 b**–**13 b**, **15 b**–**22 b** and **24 b** could be tested at the >1000 fold lower concentration of 2.8 μM. The results of the assay were analysed by PAGE (Figure 4B).

To investigate the extent of which recognition of the modified oligonucleotides by the enzyme was due to the oligonucleotide strand or whether the metal‐binding fragment had some influence on substrate recognition, 5’‐azido oligonucleotide **25** was included in the assay. Despite the fact that **25** showed moderate recognition by the enzyme (Figure 4B, lane 3), it was also clear that varying the metal‐binding fragment does influence substrate recognition. Alcohol **12 b** (Figure 4B, lane 4) and mesylate **13 b** (Figure 4B, lane 5) show minimal recognition as substrate oligonucleotide **11** was digested. Phosphate **15 b** (Figure 4B, lane 6) displays particularly effective recognition by SNM1A. The flexible linker enabled the phosphate group to bind effectively to the enzyme. As anticipated, amine **16 b** does not display effective recognition by the enzyme (Figure 4B, lane 7), whereas sulfonamide **17 b** and monosquaramide **18 b** display moderate recognition by SNM1A (Figure 4B, lane 8 and 9). Similar to the results from the fragment‐based screening approach, *N*‐hydroxysquaramides **19 b** and **20 b** displayed effective recognition by SNM1A (Figure 4B, lanes 10 and 11). The one carbon extension of the flexible linker does not seem to majorly affect enzyme recognition. Although alcohol **21 b** (Figure 4B, lane 12) and mesylates **22 b** and **24 b** (Figure 4B, lanes 13 and 14) show slightly improved levels of recognition compared to **12 b**, **13 b** (Figure 4B, lanes 4 and 5), modified oligonucleotides **21 b**, **22 b** and **24 b** are only moderately recognised by the enzyme. The modified oligonucleotides **12 b**–**13 b**, **15 b**, **22 b** and **24 b** were also tested at the lower concentration of 1.6 μM (Figure S3) but did not affect enzymatic activity at this concentration. Interestingly, when the click fragments **12 a**–**13 a**, **15 a**–**22 a** and **24 a** were tested against SNM1A at 5 mM (Figure S4), only phosphate **15 a** was recognised whereas squaramide moieties **19 a** and **20 a** were not recognised. However, incorporation of the squaramide moieties into an oligonucleotide strand enhanced their recognition as **19 b** and **20 b** were recognised at 2.8 μM.

Overall, inclusion of the oligonucleotide strand greatly increased the potency of substrate recognition, from 5 mM of the metal‐binding fragments alone to 2.8 μM when incorporated in an oligonucleotide strand. This translates to a SNM1A : metal‐binding fragment ratio of 1 : 1×10^6^ when the fragments are tested alone and a ratio of 1 : 560 when the metal binding fragments are incorporated into a nucleotide strand. The large discrepancy in potency can be rationalised by the oligonucleotide anchoring the fragment within the active site of the protein. This anchoring effect significantly increases the avidity of the molecule, blocking the active site at much lower concentrations. Incorporation of metal‐binding fragments into the 5’‐position of an oligonucleotide strand using the CuAAC reaction is an effective method of generating unnatural oligonucleotides recognised by SNM1A.

## Conclusion

To conclude, we have reported a two‐step validation process for the identification of metal‐binding fragments suitable for targeting SNM1A. This consisted of a fragment‐based screening approach followed by incorporation of fragments into oligonucleotides by click chemistry. The fragment‐based screening approach successfully identified metal‐binding fragments suitable for targeting SNM1A. This is the first time fragment‐based screening has been used to identify fragments for targeting SNM1A and demonstrates the extensive utility of a fragment‐based screening approach.

We have also shown that incorporation of these metal‐binding fragments into an oligonucleotide strand with a CuAAC reaction is an effective method for rapidly generating unnatural oligonucleotides recognised by SNM1A and screening metal‐binding fragments suitable for targeting the enzyme. Previously, the generation of modified nucleosides/oligonucleotides recognised by SNM1A required extensive synthesis of modified nucleosides or phosphoramidites, which is both time‐consuming and expensive.[^7^, ^8^] Here, we have demonstrated a reliable method of rapidly installing 5′‐modifications into oligonucleotides using the CuAAC reaction. All Cu^I^‐catalysed triazole formations attempted in this study proceeded smoothly with high conversion, thus demonstrating the wide applicability of this method. Furthermore, inclusion of the oligonucleotide strand greatly increased the potency of substrate recognition, from 5 mM of the metal‐binding fragments alone to 2.8 μM when incorporated in an oligonucleotide strand. While the unnatural triazole linkage is recognised by SNM1A, these modified oligonucleotides are not recognised as effectively as naturally linked oligonucleotides, which were previously reported as being recognised at concentrations as low as 0.08 μM.^[8]^

The strategy of peptide tethering is well established.[^24^, ^31^] This strategy involves the incorporation of fragments into peptides, thereby enhancing their binding to targeted protein pockets. Our novel approach of incorporating fragments into oligonucleotides may be similarly applied to generate modified oligonucleotides or DNA mimics with high affinity for targeted DNA damage repair enzymes. The linker between the fragment and the oligonucleotide may be optimised in terms of linker length and type to further enhance binding of target enzymes.

The two‐step validation process reported here could be applied in the future for the rapid identification of potent fragments capable of selectively targeting DNA damage‐repair enzymes. These fragments could then be incorporated into nucleoside/oligonucleotide scaffolds or combined to generate probes to study DNA damage repair. Our results demonstrate a novel synthetic methodology for rapidly installing metal‐binding fragments in the 5’‐position of oligonucleotides. This is a facile method of generating oligonucleotides with metal‐binding capabilities.

## Experimental Section

**General methods**: Chemicals were purchased from Acros Organics, Aldrich, Alfa Aesar, Carbosynth, Fisher Scientific, Fluorochem, Sigma Aldrich, Merck and TCI Chemicals and were used as purchased without further purification. THF and CH_2_Cl_2_ were dried using a PureSolv MD solvent purification system. Oxygen‐free anhydrous argon was obtained from BOC gases. Flash column chromatography was carried out using silica gel, particle size 0.04–0.063 mm, purchased from Sigma Aldrich or VWR. TLC analysis was performed on TLC Silica gel 60 F_254_ plates purchased from Merck and was visualised by UV irradiation (254 nm), potassium permanganate stain (3 g KMnO_4_, 20 g K_2_CO_3_, 300 mL H_2_O), ninhydrin stain (1.5 g ninhydrin, 5 mL AcOH, 500 mL 95 % EtOH), anisaldehyde stain (9.2 mL *p‐*methoxybenzaldehyde, 3.75 mL AcOH, 338 mL 95 % EtOH, 12.5 mL conc. H_2_SO_4_) and iodine. ^1^H and ^13^C NMR spectra were recorded on Bruker 400 MHz or 600 MHz system spectrometers in [D_6_]DMSO, CDCl_3_, CD_3_OD or [D_6_]acetone relative to residual DMSO (*δ*_H_=2.50 ppm, *δ*_C_=39.52 ppm), CDCl_3_, (*δ*_H_=7.26 ppm, *δ*_C_=77.16 ppm), CD_3_OD (*δ*_H_=3.31 ppm, *δ*_C_=49.00 ppm) or [D_6_]acetone (*δ*_H_=2.05 ppm, *δ*_C_=29.84 ppm).^[32]^ Chemical shifts (d) are reported in ppm and coupling constants are reported in Hertz accurate to 0.2 Hz. ^13^C NMR spectra are proton‐decoupled. NMR spectra were assigned using HSQC, HMBC, DEPT and TOCSY experiments. Mass spectrometry measurements were carried out on a Bruker ESI, APCI or MALDI HRMS. Infrared spectra were obtained on a Perkin Elmer spectrophotometer. Melting points were measured using a Griffin melting point apparatus and are uncorrected.

**3‐Ethoxy‐4‐(hydroxy(methyl)amino)cyclobut‐3‐ene‐1,2‐dione 5**: *N*‐Methylhydroxylamine hydrochloride (58 mg, 0.69 mmol) was suspended in EtOH (2 mL). Et_3_N (290 μL, 2.08 mmol) and diethyl squarate **2** (100 μL, 0.68 mmol) were added and the reaction mixture was stirred at 18 °C for 22 h. After this time, TLC analysis (CH_2_Cl_2_/EtOH, 4 : 1) showed the consumption of starting material (*R*_f_=0.9) and the formation of product (*R*_f_=0.6). Purification by flash column chromatography (CH_2_Cl_2_/EtOH, 98 : 2) yielded the desired product **5** as a white solid (113 mg, 97 %); mp 96–100 °C. IR (ATR) ν_max_/cm^−1^: 2981 (C−H), 2920 (C−H), 1796, 1701 (C=O), 1560 (C=O), 1499, 1545, 1242. ^1^H NMR (400 MHz, [D_6_]DMSO) *δ*=1.36 (t, *J* 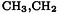 =7.1 Hz, 3H, CH_3_^Et^), 3.38 (s, 3H, CH_3_^Me^), 4.65 (q, *J* 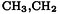 =7.1 Hz, 2H, CH_2_^Et^), 10.97 (s, 1H, OH).^13^C NMR (151 MHz, [D_6_]DMSO) *δ*=15.6 (CH_3_^Et^), 40.8 (CH_3_^Me^), 69.0 (CH_2_^Et^), 168.1 (qC), 174.2 (qC), 179.2 (CO), 185.1 (CO). HRMS (ESI^‐^): *m*/*z* calcd. 170.0459 [*M*−H]^−^, found: 170.0455.

**Di‐*tert*‐butyl pent‐4‐yn‐1‐yl phosphate 14**: 4‐Pentyn‐1‐ol **12 a** (100 μL, 1.07 mmol) was dissolved in anhydrous CH_2_Cl_2_ (8 mL) under argon. Tetrazole (3–4 % in CH_3_CN, 9.41 mL) was added and the reaction mixture was stirred on ice. Di‐*tert*‐butyl diisopropylphosphoramidite (0.54 mL, 1.71 mmol) was added dropwise on ice. The reaction mixture was warmed to 18 °C and stirred for 3 h. After this time, another portion of di‐*tert*‐butyl diisopropylphosphoramidite (0.43 mL, 1.08 mmol) was added dropwise on ice. The reaction mixture was warmed to 18 °C and stirred for another 3.5 h. After this time TLC analysis (Hex/EtOAc, 1 : 1) showed consumption of the starting material (*R*_f_=0.7) to give an intermediate product (*R*_f_=0.4). The mixture was cooled on ice and *m*‐CPBA (70–75 % purity, 689 mg) was added. The reaction mixture was warmed to 18 °C and stirred for 17 h. After this time TLC analysis (Hex/EtOAc, 1 : 1) showed disappearance of the intermediate product (*R*_f_=0.4) to give a new product (*R*_f_=0.5). The reaction was quenched by addition of 10 % (*w*/*v*) of aq. sodium thiosulfate (20 mL) and stirred at 18 °C for 1.5 h. The layers were separated and the aqueous layer was extracted with CH_2_Cl_2_ (3×30 mL). The combined organics were washed with sat. aq. NaHCO_3_ (50 mL), dried over MgSO_4_, filtered and the solvent was removed in vacuo. The crude product was purified by flash column chromatography (Hex/EtOAc, 4 : 1→1 : 1) to give the product **14** as a colourless oil (25 mg, 8 %). IR (ATR) *ν*_max_/cm^−1^: 3299 (≡C−H), 3222 (≡C−H), 2980 (C−H), 1476 (P=O), 1395, 1370 (≡C−H), 1260 (≡C−H), 1174, 1091, 1037 (P−O), 980 (C−C≡C), 919, 860, 833, 807, 742 (P−C). ^1^H NMR (400 MHz, CDCl_3_): *δ*=1.49 (s, 18H, 6×CH_3_^*t*Bu^), 1.84–1.91 (m, 2H, CH_2_), 1.96 (t, *J*_3,5_=2.6 Hz, 1H, ≡C−H), 2.33 (td, *J*_3,5_=2.6 Hz, *J*_2,3_=7.1 Hz, 2H, CH_2_), 4.05 (app. q, *J*=6.3 Hz, 2H, CH_2_) ppm. ^13^C NMR (101 MHz, CDCl_3_): *δ*=15.0 (CH_2_), 29.3 (d, *J*_C,P_=7.4 Hz, CH_2_), 30.0 (d, *J*_C,P_=4.3 Hz, CH_3_^*t*Bu^), 65.3 (d, *J*_C,P_=6.3 Hz, CH_2_), 69.0 (≡C−H), 82.3 (d, *J*_C,P_=7.0 Hz, qC^*t*Bu^), 83.4 (qC) ppm. ^31^P NMR (162 MHz, CDCl_3_): *δ*=−9.74 ppm. HRMS (ESI^+^): *m/z* calcd. 299.1383 [*M*+Na]^+^, found 299.1383

**Disodium pent‐4‐yn‐1‐yl phosphate 15 a**: A solution of TFA/CH_2_Cl_2_, 1 : 3 (0.65 mL) was added to **14** (18 mg, 65 μmol) and the pink solution was stirred at 18 °C for 1 h. After this time, TLC analysis (Hex/EtOAc, 1 : 4) showed consumption of the starting material (*R*_f_=0.73) to give the product (*R*_f_=0.04). Toluene (∼5 mL) was added and the solvent was removed in vacuo. Excess TFA was removed by coevaporation with toluene (3×3 mL). The pink residue was redissolved in CH_2_Cl_2_/CH_3_OH, 9 : 1 and eluted through Diaion resin WT01S(H) (Na form) in CH_3_OH to afford **15 a** as a white film (5.5 mg, 39 %). IR (ATR, CH_3_OH) ν_max_/cm^−1^: 3284 (≡C−H), 2957 (C−H), 1649 (P=O), 1440 (C−H), 1192, 1085, 1052 (P−O), 983 (C−C≡C), 896, 817, 782. ^1^H NMR (400 MHz, CD_3_OD): *δ*=1.81 (app. quin, *J*=6.8 Hz, 2H, CH_2_), 2.15 (t, *J*_3,5_=2.7 Hz, 1H, ≡C−H), 2.29 (td, *J*_3,5_=2.7 Hz, *J*_2,3_=7.3 Hz, 2H, CH_2_), 3.90 (app. q, *J*=6.2 Hz, 2H, CH_2_) ppm. ^13^C NMR (101 MHz, CD_3_OD): *δ*=15.8 (CH_2_), 31.5 (d, *J*_C,P_=7.8 Hz, CH_2_), 64.3 (d, *J*_C,P_=5.0 Hz, CH_2_), 69.3 (≡C−H), 84.9 (qC) ppm. ^31^P NMR (162 MHz, CD_3_OD): *δ*=3.06 ppm. HRMS (ESI^+^): *m/z* calcd. 208.9950 [*M*+H]^+^, found 208.9949

***N*** **‐(Pent‐4‐yn‐1‐yl)methanesulfonamide 17 a**: Pent‐4‐yn‐1‐amine hydrochloride **16 a** (50 mg, 0.42 mmol) was suspended in anhydrous CH_2_Cl_2_ (4 mL) under argon. Et_3_N (0.15 mL, 1.08 mmol) was added to give a colourless solution and the mixture was stirred on ice. MsCl (49 μL, 0.63 mmol) was added dropwise on ice and the reaction mixture was stirred at 0–18 °C over 18 h. After this time TLC analysis (CH_2_Cl_2_−CH_3_OH, 19 : 1) showed consumption of starting material (*R*_f_=0.1) to give the product (*R*_f_=0.7). The reaction was quenched on addition of CH_3_OH (1 mL) and the solvent was removed in vacuo. The product was purified by flash column chromatography (Hex/EtOAc, 1 : 1) to give the product **17 a** as a colourless oil (53 mg, 79 %). IR (ATR) *ν*_max_/cm^−1^: 3282 (N−H), 2929 (C−H), 1434 (C−H), 1411, 1308 (C−H), 1142, 1079 (S=O), 976 (C−C≡C), 850, 820, 762 (C−H). ^1^H NMR (400 MHz, CDCl_3_): *δ*=1.80 (app. quin, *J*=6.8 Hz, 2H, CH_2_), 2.01 (t, *J*_4,6_=2.6 Hz, 1H, ≡C−H), 2.32 (td, *J*_4,6_=2.6 Hz, *J*_3,4_ *=*6.8 Hz, 2H, CH_2_), 2.98 (s, 3H, CH_3_), 3.29 (app. q, *J*=6.2 Hz, 2H, CH_2_), 4.38 (br s, 1H, NH) ppm. ^13^C NMR (101 MHz, CDCl_3_): *δ*=15.8 (CH_2_), 28.6 (CH_2_), 40.6 (CH_3_), 42.2 (CH_2_), 69.9 (≡C−H), 82.8 (qC) ppm. HRMS (ESI^+^): *m/z* calcd. 184.0403 [*M*+Na]^+^, found 184.0410

**3‐Ethoxy‐4‐(pent‐4‐yn‐1‐ylamino)cyclobut‐3‐ene‐1,2‐dione 18 a**: Pent‐4‐yn‐1‐amine **16 a** (50 mg, 0.42 mmol) and Et_3_N (0.12 mL, 0.86 mmol) were dissolved in EtOH (4 mL). Diethyl squarate **2** (87 μL, 0.59 mmol) was added. The reaction mixture was stirred at 18 °C for 18 h. After this time, TLC analysis (CH_2_Cl_2_/CH_3_OH, 9 : 1) showed the complete consumption of starting material (*R*_f_=0.1) to give the product (*R*_f_=0.7). The solvent was removed in vacuo and the crude product was purified by flash column chromatography (Hex/EtOAc, 1 : 1) to give the desired product **18 a** as a white solid (87 mg, quant.); mp 57–58 °C. IR (ATR) *ν*_max_/cm^−1^: 3296 (N−H), 3198 (C−H), 2935 (C−H), 2117, 1698 (C=O), 1577 (C=O), 1490, 1464, 1433, 1376 (C−H), 1357, 1338, 1317, 1257, 1198, 1153, 1117, 1050, 1004 (C−C≡C), 989, 874, 852, 808, 788 (C−H). ^1^H NMR (400 MHz, CDCl_3_): *δ*=1.46 (t, *J*_1,2_=7.1 Hz, 3H, CH_3_^Et^), 1.86 (app. quin, *J*=6.8 Hz, 2H, CH_2_), 2.01 (t, *J*_9,11_=2.6 Hz, 1H, ≡C−H), 2.31 (td, *J*_9,11_=2.6 Hz, *J*_8,9_=6.9 Hz, 1H, CH_2_), 3.57–3.61 (m, 1.5H, CH_2_), 3.79 (br s, 0.5H, CH_2_), 4.76–4.79 (m, 2H, CH_2_^Et^), 5.53 (br s, 0.3H, NH), 6.54 (br s, 0.7H, NH) ppm. ^13^C NMR (135 MHz, CDCl_3_): *δ*=15.7 (CH_2_), 16.0 (CH_3_^Et^), 29.1 (CH_2_), 43.9 (CH_2_), 69.9 (C‐2, ≡C−H), 82.5 (qC), 172.6 (qC), 177.7 (qC), 183.0 (CO), 189.5 (CO) ppm. Note: **18 a** exhibits rotamers in NMR spectroscopy. HRMS (APCI^−^): *m/z* calcd. 206.0823 [*M*−H]^−^, found 206.0825

**3‐(Hydroxy(methyl)amino)‐4‐(pent‐4‐yn‐1‐ylamino)cyclobut‐3‐ene‐1,2‐dione 19 a**: Compound **18 a** (49 mg, 0.24 mmol) was dissolved in EtOH (2 mL). *N*‐Methylhydroxylamine hydrochloride (32 mg, 0.38 mmol) and Et_3_N (0.1 mL, 0.72 mmol) were added, and the reaction mixture was stirred at 18 °C for 19 h. After this time, TLC analysis (CH_2_Cl_2_−CH_3_OH, 9 : 1) showed complete consumption of the starting material (*R*_f_=0.7) to give the product (*R*_f_=0.3). The solvent was removed in vacuo, and the product was purified by flash column chromatography (CH_2_Cl_2_/CH_3_OH, 9 : 1) to give **19 a** as a white solid (40 mg, 82 %); mp 130–132 °C. IR (ATR) ν_max_/cm^−1^: 3274 (N−H), 3066 (O−H), 2772 (C−H), 1799, 1642 (C=O), 1527 (C=O), 1447, 1421, 1397, 1351 (C−H), 1275, 1234, 1140, 1120, 1012 (C−C≡C), 885, 866, 821, 761 (C−H). ^1^H NMR (400 MHz, CDCl_3_): *δ*=1.73 (app. quin, *J*=7.1 Hz, 2H, CH_2_), 2.18 (td, *J*_8,10_=2.7 Hz, *J*_7,8_=7.3 Hz, 2H, CH_2_), 2.78 (t, *J*_8,10_=2.7 Hz, 1H, ≡C−H), 3.34 (s, 3H, CH_3_), 3.52 (app. q, *J*=6.7 Hz, 2H, CH_2_), 7.69 (br s, 1H, NH), 10.57 (br s, 1H, OH) ppm. ^13^C NMR (135 MHz, CDCl_3_): *δ*=15.0 (CH_2_), 29.7 (CH_2_), 40.8 (CH_3_), 42.8 (CH_2_), 71.5 (≡C−H), 83.8 (qC), 165.9 (qC), 167.1 (qC), 178.3 (CO), 180.1 (CO) ppm. HRMS (ESI^‐^): *m/z* calcd. 207.0775 [*M*−H]^−^, found 207.0776

**3‐(Hydroxyamino)‐4‐(pent‐4‐yn‐1‐ylamino)cyclobut‐3‐ene‐1,2‐dione 20 a**: Compound **18 a** (54 mg, 0.26 mmol), hydroxylamine hydrochloride (29 mg, 0.42 mmol) and Et_3_N (0.11 mL, 0.79 mmol) were dissolved in EtOH and stirred at 18 °C for 18 h. After this time, TLC analysis showed the complete consumption of starting material (*R*_f_=0.7, CH_2_Cl_2_/CH_3_OH, 9 : 1) to give the product (*R*_f_=0.2, CH_2_Cl_2_/CH_3_OH, 9 : 1). The solvent was removed in vacuo, and the crude product was purified by flash column chromatography (CH_2_Cl_2_/CH_3_OH, 17 : 3) to give the product **20 a** as a pale red solid (40 mg, 78 %); mp 140 °C (decomp.). IR (ATR) *ν*_max_/cm^−1^: 3292 (N−H), 3176 (O−H), 2835 (C−H), 1802, 1647 (C=O), 1615, 1543 (C=O), 1475, 1447, 1420, 1350, 1309 (C−H), 1254, 1125, 1103, 1062, 1031, 994 (C−C≡C), 866, 838, 767, 724 (C−H), 664, 639, 612. ^1^H NMR (600 MHz, [D_6_]DMSO): *δ*=1.72 (app. quin, *J*=7.2 Hz, 2H, CH_2_), 2.18 (td, *J*_7,9_=2.6 Hz, *J*_6,7_=7.3 Hz, 2H, CH_2_), 2.78 (t, *J*_7,9_=2.6 Hz, 1H, ≡C−H), 3.51 (br s, 2H, H‐5), 7.57 (br s, 1H, NH), 9.68 (s, 1H, NHOH), 10.59 (br s, 1H, NHOH) ppm. ^13^C NMR (135 MHz, [D_6_]DMSO): *δ*=15.0 (CH_2_), 29.7 (CH_2_), 42.9 (CH_2_), 71.5 (≡C−H), 83.8 (qC), 166.6 (qC), 166.9 (qC), 178.9 (CO), 180.7 (CO) ppm. HRMS (ESI^−^): *m/z* calcd. 193.0619 [*M*−H]^−^, found 193.0622

**General Procedure: Preparation of modified nucleosides 12 b–13 b**, **15 b–22 b and 24 b using the CuAAC reaction**: H_2_O was degassed with argon prior to carrying out the reaction. Alkyne‐containing compounds **12 a**–**13 a**, **15 a**–**22 a** and **24 a** were dried under high vacuum over phosphorous pentoxide prior to use in CuAAC reactions and were added to the reaction mixture as a solution in H_2_O (0.04 or 0.05 M, 0–40 % DMSO). 5’‐Azido oligonucleotide **25** (20 μL, 1.5 mM in H_2_O) was added to a solution of alkyne‐containing compound **12 a**–**13 a**, **15 a**–**22 a** and **24 a** (35 μL) and the mixture was degassed with argon. A solution of Cu^I^ catalyst was prepared by combining tris‐hydroxypropyltriazole ligand (THPTA; 1.6 mg, 3.68 μmol) with sodium ascorbate solution (30 μL, 505 mM in H_2_O) followed by a solution of CuSO_4_ ⋅ 5H_2_O (15 μL, 100 mM in H_2_O) and the catalyst solution was degassed with argon. The catalyst was immediately added to the solution of 5’‐azido oligonucleotide **25** and alkyne containing compound and degassed with argon. The reaction mixture was incubated at 18 °C for 2 h with shaking at 200 rpm. After this time, the reaction was quenched by addition of a solution of EDTA (2 μL, 0.5 M in H_2_O, pH 8) and desalted using NAP‐5 column (GE Healthcare) according to manufacturer instructions to give modified oligonucleotides **12 b**–**13 b**, **15 b**–**22 b** and **24 b**.

**Gel electrophoresis assay**: Truncated human SNM1A (698‐1040) was stored as a 1.0 μM (0.04 mg/mL) solution in reaction buffer (20 mM HEPES‐KOH pH 7.5, 50 mM KCl, 10 mM MgCl_2_, 0.05 % Triton‐X, 0.1 mg/mL BSA, 5 % glycerol, 0.5 mM DTT). All incubations were carried out with gentle shaking (80 rpm). Fragments **1**–**8** were dried under high vacuum over phosphorous pentoxide prior to use in the assay. Fragments **1**–**8** (5 mM in reaction) or oligonucleotides **25**, **12 b**–**13 b**, **15 b**–**22 b** and **24 b** (2.8 μM in reaction) were treated with SNM1A (50 fmol) in reaction buffer (10 μL, for fragment screening 4 % DMSO was used) on ice, and then incubated at 37 °C for 5 min. A solution of the oligonucleotide substrate **11** (1 μL, 0.8 pmol/μL) was added and each reaction was incubated at 37 °C for a further 60 min. The reactions were stopped by addition of 2 μL of stop solution (95 % formamide, 10 mM EDTA) followed by heating at 95 °C for 3 min. Digested oligonucleotides were separated on 15 % acrylamide 6.5 M urea gels (2.9 g urea, 2.7 mL 40 % acrylamide/bisacrylamide 25 : 1, 0.7 mL 10× TBE (0.9 M Tris, 0.9 M boric acid, 0.02 M EDTA pH 8.0), 1.3 mL H_2_O) in 1x TBE at 150 V for between 75 and 90 min, alongside bromophenol blue and xylene cyanol as markers for 8 and 28 nt, respectively. The gels were imaged by using a Typhoon FLA 9500.

## Conflict of interest

The authors declare no conflict of interest.

1

## Biographical Information

*Joanna McGouran is the Schuler Assistant Professor In Translational Organic Chemistry at Trinity College Dublin, Ireland. She holds a PhD from the University of Oxford focused on carbohydrate chemistry. Her postdoctoral work at the University of Oxford included activity‐based protein profiling and oligonucleotide modification. Research in her group focuses on creating chemical tools through biomolecule modification including nucleic acids, peptides, and proteins*.

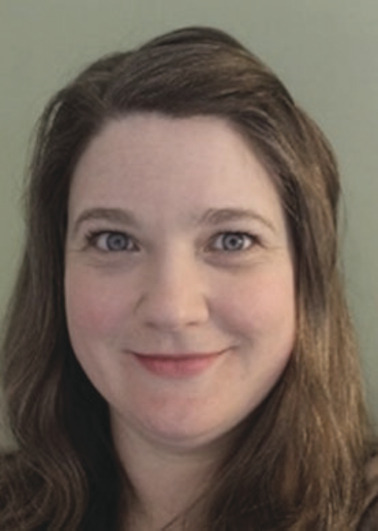

## Biographical Information

*Ellen Fay is a PhD student studying organic chemistry and chemical biology in the School of Chemistry, Trinity College Dublin under the supervision of Prof. Joanna McGouran. Ellen completed her BA (mod) in chemistry in Trinity College Dublin in 2019. Ellen's PhD research focuses on the development of molecular tools to study DNA damage‐repair enzymes. These poorly understood enzymes play a key role in resistance to cancer chemotherapy*.

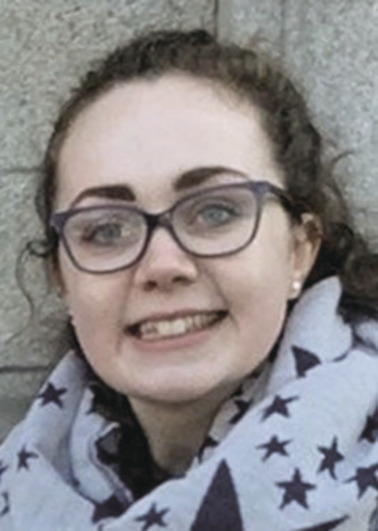

## Supporting information

As a service to our authors and readers, this journal provides supporting information supplied by the authors. Such materials are peer reviewed and may be re‐organized for online delivery, but are not copy‐edited or typeset. Technical support issues arising from supporting information (other than missing files) should be addressed to the authors.

Supporting Information

## Data Availability

The data that support the findings of this study are available in the supplementary material of this article.

## References

[1] G. Bahr , L. J. Gonzalez , A. J. Vila , Chem. Rev. 2021, 121, 7957–8094. 34129337 10.1021/acs.chemrev.1c00138 PMC9062786

[2a] U. B. Abdullah , J. F. McGouran , S. Brolih , D. Ptchelkine , A. H. El-Sagheer , T. Brown , P. J. McHugh , EMBO J. 2017, 36, 2047–2060; 28607004 10.15252/embj.201796664 PMC5510000

[2b] E. M. Tacconi , S. Badie , G. De Gregoriis , T. Reisländer , X. Lai , M. Porru , C. Folio , J. Moore , A. Kopp , J. B. Torres , EMBO Mol. Med. 2019, 11, e9982. 31273933 10.15252/emmm.201809982 PMC6609913

[3] J. Hejna , S. Philip , J. Ott , C. Faulkner , R. Moses , Nucleic Acids Res. 2007, 35, 6115–6123. 17804464 10.1093/nar/gkm530 PMC2094091

[4] B. Sengerová , C. K. Allerston , M. Abu , S. Y. Lee , J. Hartley , K. Kiakos , C. J. Schofield , J. A. Hartley , O. Gileadi , P. J. McHugh , J. Biol. Chem. 2012, 287, 26254–26267. 22692201 10.1074/jbc.M112.367243 PMC3406710

[5] C. K. Allerston , S. Y. Lee , J. A. Newman , C. J. Schofield , P. J. McHugh , O. Gileadi , Nucleic Acids Res. 2015, 43, 11047–11060. 26582912 10.1093/nar/gkv1256 PMC4678830

[6] S. Y. Lee , J. Brem , I. Pettinati , T. D. Claridge , O. Gileadi , C. J. Schofield , P. J. McHugh , Chem. Commun. 2016, 52, 6727–6730. 10.1039/c6cc00529b PMC5063058 27121860

[7] M. Berney , M. T. Manoj , E. M. Fay , J. F. McGouran , ChemMedChem 2022, 17, e202100603. 34905656 10.1002/cmdc.202100603

[8] E. M. Dürr , W. Doherty , S. Y. Lee , A. H. El-Sagheer , A. Shivalingam , P. J. McHugh , T. Brown , J. F. McGouran , ChemistrySelect 2018, 3, 12824–12829. 31414040 10.1002/slct.201803375 PMC6685075

[9] A. F. Sandahl , T. J. Nguyen , R. A. Hansen , M. B. Johansen , T. Skrydstrup , K. V. Gothelf , Nat. Commun. 2021, 12, 1–7. 33958587 10.1038/s41467-021-22945-z PMC8101336

[10] B. Buzon , R. A. Grainger , C. Rzadki , S. Y. M. Huang , M. Junop , ACS Omega 2021, 6, 9352–9361. 33869915 10.1021/acsomega.0c03528 PMC8047731

[11a] M. Berney , W. Doherty , W. T. Jauslin , M. T. Manoj , E.-M. Dürr , J. F. McGouran , Bioorg. Med. Chem. 2021, 46, 116369; 34482229 10.1016/j.bmc.2021.116369 PMC8607331

[11b] E.-M. Dürr , J. F. McGouran , Molecules 2021, 26, 320; 33435514 10.3390/molecules26020320 PMC7827217

[11c] W. Doherty , E. M. Durr , H. T. Baddock , S. Y. Lee , P. J. McHugh , T. Brown , M. O. Senge , E. M. Scanlan , J. F. McGouran , Org. Biomol. Chem. 2019, 17, 8094–8105. 31380542 10.1039/c9ob01133a PMC6984127

[12] P. J. Hajduk , J. Greer , Nat. Rev. Drug Discovery 2007, 6, 211–219. 17290284 10.1038/nrd2220

[13] UCSF Chimera, https://www.rbvi.ucsf.edu/chimera, accessed February 2023.

[14] D. A. Erlanson , R. S. McDowell , T. O′Brien , J. Med. Chem. 2004, 47, 3463–3482. 15214773 10.1021/jm040031v

[15] M. Bon , A. Bilsland , J. Bower , K. McAulay , Mol. Oncol. 2022, 16, 3761-3777 . 35749608 10.1002/1878-0261.13277 PMC9627785

[16] D. M. Wilson III , A. M. Deacon , M. A. Duncton , P. Pellicena , M. M. Georgiadis , A. P. Yeh , A. S. Arvai , D. Moiani , J. A. Tainer , D. Das , Prog. Biophys. Mol. Biol. 2021, 163, 130–142. 33115610 10.1016/j.pbiomolbio.2020.10.005 PMC8666131

[17] H. Guo , K. Cheng , Y. Gao , W. Bai , C. Wu , W. He , C. Li , Z. Li , Bioorg. Med. Chem. 2020, 28, 115437. 32229085 10.1016/j.bmc.2020.115437

[18a] F. Müggenburg , S. Müller , The Chemical Record 2022, 22, e202100322; 35189013 10.1002/tcr.202100322

[18b] A. H. El-Sagheer , T. Brown , Chem. Soc. Rev. 2010, 39, 1388–1405; 20309492 10.1039/b901971p

[18c] N. Z. Fantoni , A. H. El-Sagheer , T. Brown , Chem. Rev. 2021, 121, 7122–7154. 33443411 10.1021/acs.chemrev.0c00928

[19] S. Berndl , N. Herzig , P. Kele , D. Lachmann , X. Li , O. S. Wolfbeis , H.-A. Wagenknecht , Bioconjugate Chem. 2009, 20, 558–564. 10.1021/bc8004864 19220008

[20] P. M. Gramlich , S. Warncke , J. Gierlich , T. Carell , Angew. Chem. Int. Ed. 2008, 47, 3442–3444. 10.1002/anie.200705664 18383495

[21] T. Lauria , C. Slator , V. McKee , M. Müller , S. Stazzoni , A. L. Crisp , T. Carell , A. Kellett , Chem. Eur. J. 2020, 26, 16782–16792. 32706904 10.1002/chem.202002860

[22a] A. Nuzzi , A. Massi , A. Dondoni , QSAR Comb. Sci. 2007, 26, 1191–1199;

[22b] V. Madhuri , V. A. Kumar , Nucleosides Nucleotides Nucleic Acids 2012, 31, 97–111; 22303990 10.1080/15257770.2011.644100

[22c] A. M. Varizhuk , D. N. Kaluzhny , R. A. Novikov , A. O. Chizhov , I. P. Smirnov , A. N. Chuvilin , O. N. Tatarinova , G. Y. Fisunov , G. E. Pozmogova , V. L. Florentiev , J. Org. Chem. 2013, 78, 5964–5969; 23724994 10.1021/jo400651k

[22d] A. P. Sanzone , A. H. El-Sagheer , T. Brown , A. Tavassoli , Nucleic Acids Res. 2012, 40, 10567–10575; 22904087 10.1093/nar/gks756 PMC3488222

[22e] A. Varizhuk , A. Chizhov , V. Florentiev , Bioorg. Chem. 2011, 39, 127–131; 21474159 10.1016/j.bioorg.2011.03.002

[22f] Y. R. Baker , D. Traore , P. Wanat , A. Tyburn , A. H. El-Sagheer , T. Brown , Tetrahedron 2020, 76, 130914;

[22g] A. Shivalingam , A. E. Tyburn , A. H. El-Sagheer , T. Brown , J. Am. Chem. Soc. 2017, 139, 1575–1583; 28097865 10.1021/jacs.6b11530

[22h] S. Chandrasekhar , P. Srihari , C. Nagesh , N. Kiranmai , N. Nagesh , M. M. Idris , Synthesis 2010, 2010, 3710–3714;

[22i] R. Lucas , R. Zerrouki , R. Granet , P. Krausz , Y. Champavier , Tetrahedron 2008, 64, 5467–5471;

[22j] H. Isobe , T. Fujino , N. Yamazaki , M. Guillot-Nieckowski , E. Nakamura , Org. Lett. 2008, 10, 3729–3732; 18656947 10.1021/ol801230k

[22k] S. Epple , A. Modi , Y. R. Baker , E. Wȩgrzyn , D. Traoré , P. Wanat , A. E. Tyburn , A. Shivalingam , L. Taemaitree , A. H. El-Sagheer , J. Am. Chem. Soc. 2021, 143, 16293–16301. 34546729 10.1021/jacs.1c08057 PMC8499026

[23a] C. N. Birts , A. P. Sanzone , A. H. El-Sagheer , J. P. Blaydes , T. Brown , A. Tavassoli , Angew. Chem. Int. Ed. 2014, 53, 2362–2365; 10.1002/anie.201308691 PMC4016740 24452865

[23b] A. H. El-Sagheer , A. P. Sanzone , R. Gao , A. Tavassoli , T. Brown , Proc. Natl. Acad. Sci. USA 2011, 108, 11338–11343; 21709264 10.1073/pnas.1101519108 PMC3136318

[23c] M. Kukwikila , N. Gale , A. H. El-Sagheer , T. Brown , A. Tavassoli , Nat. Chem. 2017, 9, 1089–1098. 29064492 10.1038/nchem.2850

[24] A. M. Beekman , M. M. Cominetti , S. J. Walpole , S. Prabhu , M. A. O′Connell , J. Angulo , M. Searcey , Chem. Sci. 2019, 10, 4502–4508. 31057779 10.1039/c9sc00059c PMC6482886

[25a] H. Ma , J. B. Murray , H. Luo , X. Cheng , Q. Chen , C. Song , C. Duan , P. Tan , L. Zhang , J. Liu , RSC Med. Chem. 2022, 13, 1341-1349 ; 36426238 10.1039/d2md00197g PMC9667776

[25b] A. Gironda-Martínez , E. J. Donckele , F. Samain , D. Neri , ACS Pharmacol. Transl. Sci. 2021, 4, 1265–1279. 34423264 10.1021/acsptsci.1c00118 PMC8369695

[26] J. A. Jacobsen , J. L. M. Jourden , M. T. Miller , S. M. Cohen , Biochim. Biophys. Acta Mol. Cell Res. 2010, 1803, 72–94. 10.1016/j.bbamcr.2009.08.006 19712708

[27] I. Ivancová , R. Pohl , M. Hubálek , M. Hocek , Angew. Chem. Int. Ed. 2019, 58, 13345–13348. 10.1002/anie.201906737 PMC6771961 31328344

[28a] V. K. Sharma , S. K. Singh , P. M. Krishnamurthy , J. F. Alterman , R. A. Haraszti , A. Khvorova , A. K. Prasad , J. K. Watts , Chem. Commun. 2017, 53, 8906–8909; 10.1039/c7cc04092j 28736781

[28b] A. H. El-Sagheer , T. Brown , Chem. Sci. 2014, 5, 253–259;

[28c] A. Dallmann , A. H. El-Sagheer , L. Dehmel , C. Mügge , C. Griesinger , N. P. Ernsting , T. Brown , Chem. Eur. J. 2011, 17, 14714–14717. 22131102 10.1002/chem.201102979

[29] I. Delso , J. Valero-Gonzalez , F. Gomollón-Bel , J. Castro-López , W. Fang , I. Navratilova , D. M. van Aalten , T. Tejero , P. Merino , R. Hurtado-Guerrero , ChemMedChem 2018, 13, 128–132. 29164827 10.1002/cmdc.201700720

[30] K. Singh , C. J. Fennell , E. A. Coutsias , R. Latifi , S. Hartson , J. D. Weaver , Chem 2018, 4, 124–137.

[31] A. E. Modell , F. Marrone III , N. R. Panigrahi , Y. Zhang , P. S. Arora , J. Am. Chem. Soc. 2022, 144, 1198–1204. 35029987 10.1021/jacs.1c09666 PMC8959088

[32] H. E. Gottlieb , V. Kotlyar , A. Nudelman , J. Org. Chem. 1997, 62, 7512–7515. 11671879 10.1021/jo971176v

