# Supplementary material for: Two‐Step Validation Approach for Tools To Study the DNA Repair Enzyme SNM1A
Source: Chembiochem. 2023 Jun 2;24(13):e202200756. doi: 10.1002/cbic.202200756 (PMC10962688; doi:10.1002/cbic.202200756)
Supplement: Supplementary file 1 — Supporting Information [file CBIC-24-0-s001.pdf]

ChemBioChem

Supporting Information

Two-Step Validation Approach for Tools To Study the DNA Repair Enzyme SNM1A

Ellen M. Fay, Ailish Newton, Mark Berney, Afaf H. El-Sagheer, Tom Brown, and Joanna F. McGouran*

1. Supplemental synthetic procedures 2

2. Synthesis of oligonucleotides 7

3. Biological evaluation – supplementary figures 7

4. NMR Spectra 9

5. Mass spectra of modified oligonucleotides 24

6. References.. 27

1. Supplemental synthetic procedures

3,4-bis((2-hydroxyethyl)amino)cyclobut-3-ene-1,2-dione 4: [1]

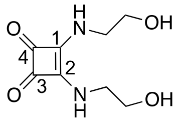

4

Diethyl squarate **2** (87 μL, 0.59 mmol) was dissolved in EtOH (3 mL). Et_3N (90 μL, 0.65 mmol) and ethanolamine (39 μL, 0.65 mmol) were added. After 5 min at rt a white precipitate formed. The reaction mixture was stirred at rt for 5 h. After this time, TLC analysis (CH_2Cl_2-EtOH, 3:2) showed the complete consumption of starting material (R_f = 0.9) and the formation of product (R_f = 0.6). Purification by flash column chromatography (CH_2Cl_2-EtOH, 3:2) yielded the product **4** as a white solid (117 mg, 99%); mp 198 ˚C

IR (ATR) ν_{max}/cm^{-1}: 3418 (N-H), 3169 (O-H), 2962 (C-H), 1799, 1560 (C=O), 1424, 1340, 1015.

^{1}H NMR (600 MHz, DMSO-*d6*) δ = 3.50-3.56 (m, 8H, 4 x CH_2), 4.90 (br s, 2H, OH), 7.56 (br s, 2H, NH) ppm.

^{13}C NMR (151 MHz, DMSO-*d6*) δ = 45.8 (CH_2), 60.8 (CH_2), 167.9 (C-1, C-2), 182.5 (C-3, C-4) ppm.

HRMS (ESI^-): m/z calc. 199.0724 $[M-H]^-$, found: 199.0729.

***N*-Methyl-benzhydroxamic acid 7:[2]**

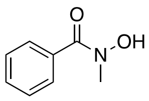

7

N-Methyl hydroxylamine hydrochloride (430 mg, 5.12 mmol) and K_2CO_3 (1.18 g, 8.54 mmol) were dissolved in H_2O (16 mL). EtOAc (32 mL) was added, and the mixture was cooled to 0 ˚C. Benzoyl chloride **10** (0.165 mL, 1.42 mmol) was added dropwise and the solution was stirred at rt for 21 h. After this time, TLC analysis (CH_2Cl_2-CH_3OH, 19:1) showed complete consumption of starting material (R_f = 0.8) to give the product (R_f = 0.7). The reaction mixture was diluted with EtOAc (50 mL) and the organic layers were washed with H_2O (30 mL), brine (30 mL), and sat. aq. $NaHCO_3$ (30 mL). The aqueous layer was extracted with EtOAc (20 mL). The combined organic layers were dried over Na_2SO_4, filtered and concentrated *in vacuo,* resulting in a yellow oil. The product was purified using flash column

chromatography (CH_2Cl_2-CH_3OH, 19:1). The product was redissolved in CH_3CN (15 mL), washed with hexane (3 x 10 mL) and concentrated to give the product **7** as a brown oil (129 mg, 60%).

IR (ATR) ν_{max}/cm^{-1}: 3147 (N-H), 3009 (O-H), 2866 (C-H), 1597 (C=O), 1570 (C=C), 1429 (C-N), 1387 (C-H), 1027 (N-O), 784 (C-H).

^{1}H NMR (400 MHz, DMSO-*d6*): δ = 3.24 (s, 3H, CH_3^{Me}), 7.39-7.45 (m, 3H, $Bz^{ortho, para}$), 7.60 (app. d, *J* = 7.1 Hz, Bz^{meta}), 9.99 (br s, 1H, OH) ppm.

^{13}C NMR (105 MHz, DMSO-*d6*): δ = 37.3 (CH_3^{Me}), 127.8 (Bz^{ortho}), 128.3 (Bz^{meta}), 130.0 (Bz^{para}), 134.9 (Bz^{qC}), 170.0 (CO) ppm.

HRMS (ESI^-): *m/z* calc. 150.0561 $[M-H]^-$, found. 150.0563

Spectroscopic data are in agreement with that reported in the literature.[2]

Benzhydroxamic acid 8:[3]

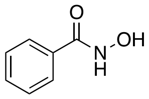

8

Hydroxylamine hydrochloride (350 mg, 5.12 mmol) and K_2CO_3 (1.18 g, 8.54 mmol) were dissolved in H_2O (16 mL). EtOAc (32 mL) was added, and the mixture was cooled to 0 ˚C. Benzoyl chloride **10** (0.165 mL, 1.42 mmol) was added dropwise and the solution was stirred at 18 ˚C for 16 h. After this time, TLC analysis (Hexane-EtOAc, 1:1) showed complete consumption of starting material (R_f = 0.9) to give the product (R_f = 0.1). The reaction mixture was diluted with EtOAc (50 mL). The organic layer washed with H_2O (30 mL), brine (30 mL), and sat. aq. $NaHCO_3$ (30 mL). The aqueous layer was extracted with EtOAc (3 x 20 mL). The combined organic layers were dried with Na_2SO_4, filtered and concentrated *in vacuo*. The crude product was partially purified using flash column chromatography (CH_2Cl_2-CH_3OH, 19:1). The product was redissolved in CH_3CN (15 mL), washed with hexane (3 x 10 mL) and the solvent was removed *in vacuo* to give the product **8** as an orange powder (89 mg, 46%); mp 124-128 ˚C. (lit.[3] 125-126 ˚C)

IR (ATR) ν_{max}/cm^{-1}: 3294 (N-H), 3181 (O-H), 2731 (C-H), 1644 (C=O), 1629 (C=C), 1453 (C-N), 1327 (C-H), 897 (N-O), 810 (C-H)

^{1}H NMR (400 MHz, CD_3OD): δ = 7.43-7.47 (m, 2H, Bz^{meta}), 7.51-7.56 (m, 1H, Bz^{para}), 7.73-7.75 (m, 2H, Bz^{ortho}) ppm.

^{13}C NMR (135 MHz, acetone-*d6*): δ = 127.8 (Bz^{para}), 129.3 (Bz^{meta}), 132.3 (Bz^{ortho}), 133.2 (Bz^{qC}), 165.9 (CO) ppm.

HRMS (ESI^-): *m/z* calc. 136.0404 $[M-H]^-$, found. 136.0405

Spectroscopic data are in agreement with that reported in the literature.[3]

Pent-4-yn-1-yl methanesulfonate 13a:[4]

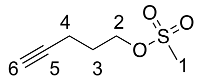

13a

4-Pentyn-1-ol **12a** (100 μL, 1.07 mmol) and Et_3N (0.18 mL, 1.29 mmol) were dissolved in anhydrous CH_2Cl_2 (10 ml) under argon. MsCl (91 μL, 1.18 mmol) was added dropwise on ice. The reaction mixture was brought to 18 ˚C and stirred for 3 h. Et_3N (0.20 mL, 1.43 mmol) was added and MsCl (100 μL, 1.30 mmol) was added on ice. The reaction mixture was brought to 18 ˚C and stirred for another 20 h. After this time, TLC analysis (CH_2Cl_2-CH_3OH, 19:1) showed complete consumption of starting material (R_f = 0.6) to give the product (R_f = 0.9). H_2O (10 mL) was added, and the product was extracted with CH_2Cl_2 (2 x 10 mL). The combined organics were dried over $MgSO_4$, filtered and the solvent was removed *in vacuo.* The crude product was purified *via* flash column chromatography (Hex-CH_2Cl_2, 1:1) to give **13a** as a colourless oil (182 mg, quant.).

IR (ATR) ν_{max}/cm^{-1}: 3288 (≡C-H), 2941 (C-H), 1469, 1436 (C-H), 1347, 1332 (C-H), 1169, 1088 (S=O), 1012, 967 (C-C≡C), 923, 826, 770, 723 (C-H).

^{1}H NMR (400 MHz, $CDCl_3$): δ = 1.96 (app. quin, *J* = 6.5 Hz, 2H, H-3), 2.01 (t, $J_{4,6}$ = 2.7 Hz, 1H, H-6), 2.37 (td, $J_{4,6}$ = 2.7 Hz, $J_{3,4}$ = 6.8 Hz, 2H, H-4), 3.03 (s, 3H, H-1) and 4.36 (t, $J_{2,3}$ = 6.1 Hz, 2H, H-2) ppm.

^{13}C NMR (101 MHz, $CDCl_3$): δ = 14.8 (C-4), 27.9 (C-3), 37.4 (C-1), 68.4 (C-2), 69.9 (C-6) and 82.2 (C-5) ppm.

Spectroscopic data are in agreement with that reported in the literature.[4]

Hex-5-yn-1-yl methanesulfonate 22a:[5]

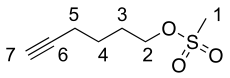

22a

5-Hexyn-1-ol **21a** (110 μL, 1.02 mmol) and Et_3N (170 μL, 1.22 mmol) were dissolved in anhydrous CH_2Cl_2 (10 mL) under argon. MsCl (87 uL, 1.1 mmol) was added dropwise at 0 ˚C. The reaction mixture was stirred from 0 ˚C – 18 ˚C over 20 h. After this time TLC analysis (CH_2Cl_2-CH_3OH, 9:1) showed consumption of starting material (R_f = 0.7) to give the product (R_f = 0.9). The reaction mixture was washed with 0.05 M aq. HCl (20 mL) and sat. aq. $NaHCO_3$ (20 mL). The combined aqueous layers were extracted with CH_2Cl_2 (2 x 20 mL) and EtOAc (2 x 20 mL). The combined organic layers were dried over $MgSO_4$, filtered and the solvent was removed *in vacuo*. The crude product was purified *via* flash column chromatography (CH_2Cl_2-CH_3OH, 19:1) to give the product **22a** as a colourless oil (147 mg, 82%).

IR (ATR) ν_{max}/cm^{-1}: 3288 (≡C-H), 2944 (C-H), 1435, 1347 (C-H), 1332, 1168, 1013 (S=O), 973 (C-C≡C), 928, 870, 819, 749 (C-H).

^{1}H NMR (400 MHz, $CDCl_3$): δ = 1.63-1.70 (m, 2H, H-4), 1.86-1.93 (m, 2H, H-3), 1.97 (t, $J_{5,7}$ = 2.6 Hz, 1H, H-7), 2.26 (td, $J_{5,7}$ = 2.6 Hz, $J_{4,5}$ = 7.0 Hz, 2H, H-5), 3.01 (s, 3H, H-1) and 4.27 (t, $J_{2,3}$ = 6.4 Hz, 2H, H-2) ppm.

^{13}C NMR (101 MHz, $CDCl_3$): δ = 18.0 (C-5), 24.4 (C-4), 28.2 (C-3), 37.6 (C-1), 69.3 (C-7), 69.5 (C-2) and 83.5 (C-6) ppm.

Spectroscopic data are in agreement with that reported in the literature.[5]

2-(Prop-2-yn-1-yloxy)ethyl methanesulfonate 24a:[6]

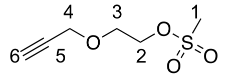

24a

Ethylene glycol **23** (0.18 mL, 3.22 mmol) was dissolved in anhydrous THF (16 ml) under argon. NaH (60% in mineral oil, 258 mg, 6.44 mmol) was added on ice and the reaction mixture was stirred on ice for 40 min. Propargyl bromide (80% in toluene, 0.52 mL, 4.83 mmol) was added dropwise on ice and the reaction mixture was heated at 50 ˚C for 17 h. After this time TLC analysis (Hex-EtOAc, 1:1) showed

consumption of starting material (R_f = 0.1) to give an intermediate (R_f = 0.3). The reaction was cooled to 18 ˚C and quenched with addition of TFA (0.5 mL). The reaction mixture was placed on ice and Et_3N (2.3 mL, 16.5 mmol) was added. MsCl (0.37 mL, 4.78 mmol) was added dropwise on ice. The reaction mixture was stirred at 0 ˚C – 18 ˚C over 24 h. After this time, TLC analysis (Hex-EtOAc, 1:1) showed consumption of the intermediate (R_f = 0.3) to give the product (R_f = 0.5). The reaction was quenched by addition of H_2O (10 mL) and the product was extracted with CH_2Cl_2 (2 x 10 mL). The combined organics were dried over $MgSO_4$, filtered and the solvent was removed *in vacuo*. The crude product was purified *via* flash column chromatography (Hex-EtOAc, 1:1) to give desired product **24a** as a pale yellow oil (37 mg, 6%).

IR (ATR) ν_{max}/cm^{-1}: 3281(≡C-H), 2929 (C-H), 1455 (C-H), 1341 (C-H), 1170, 1105 (S=O), 1018, 995, 971 (C-C≡C), 915, 836, 800

^{1}H NMR (400 MHz, $CDCl_3$): δ = 2.47 (t, $J_{4,6}$ = 2.4 Hz, 1H, H-6), 3.07 (s, 3H, H-1), 3.80-3.83 (m, 2H, H-3), 4.22 (d, $J_{4,6}$ = 2.4 Hz, 2H, H-4), 4.39-4.41 (m, 2H, H-2) ppm.

^{13}C NMR (101 MHz, $CDCl_3$): δ = 37.9 (C-1), 58.6 (C-4), 67.6 (C-3), 68.8 (C-2), 75.4 (C-6), 78.9 (C-5) ppm.

HRMS (ESI$^+$): *m/z* calc. 201.0192 [M+Na]$^+$, found 201.0187

Spectroscopic data are in agreement with that reported in the literature.[6]

2. Synthesis of oligonucleotides

5’-azido containing oligonucleotide **25** and fluorescent substrate oligonucleotide **11** were synthesised using standard solid phase oligonucleotide synthesis.[7] Fluorescent substrate oligonucleotide **11** was phosphorylated using T4PNK (New England Biolabs) according to the manufacturer’s instructions.

Sequence of 5’-azido oligonucleotide **25**: 5'-(azideT)AG CAG TCA GTC AGT CAT GC-3'

Sequence of fluorescent substrate oligonucleotide **11**: 5’-XTAG CAG TCA GTC AGT CAT CGY-3’

X = Thymidine 5’-phosphate

Y = Cy3

3. Biological evaluation – supplementary figures

Screening of fragments 1-8 for recognition by SNM1A at 5 mM in duplicate

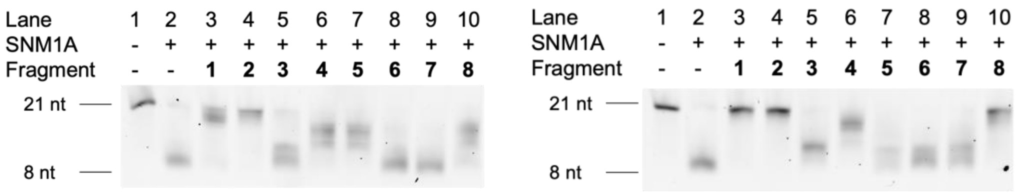

Figure S1. Evaluation of recognition of fragments **1-8** by SNM1A. Digestion of fluorescent substrate **11** (0.8 pmol) after incubation with SNM1A (50 fmol) for 60 min at 37 °C following 5 min preincubation with fragments **1-8** (5 mM). nt = nucleotides Sequence of fluorescent substrate oligonucleotide **11**: 5’-XTAG CAG TCA GTC AGT CAT CGY-3’ X = Thymidine 5’-phosphate, Y = Cy3

Screening of modified oligonucleotides 12b-13b, 15b-22b and 24b for recognition by SNM1A at 2.8 μM in duplicate

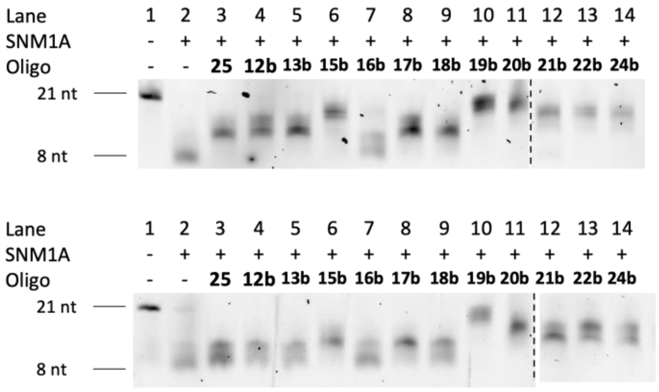

Figure S2. Evaluation of recognition of modified oligonucleotides **12b-13b, 15b-22b, 24b** by SNM1A. Digestion of fluorescent substrate **11** (0.8 pmol) after incubation with SNM1A (50 fmol) for 60 min at 37 °C following 5 min preincubation with oligonucleotides **12b-13b, 15b-22b, 24b** (2.8 μM). nt = nucleotides

Screening of modified oligonucleotides 12b-13b, 15b-22b and 24b for recognition by SNM1A at 1.6 μM

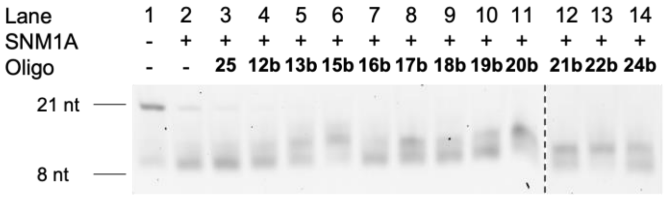

Figure S3. Evaluation of recognition of modified oligonucleotides **12b-13b, 15b-22b, 24b** by SNM1A. Digestion of fluorescent substrate **11** (0.8 pmol) after incubation with SNM1A (50 fmol) for 60 min at 37 °C following 5 min preincubation with oligonucleotides **12b-13b, 15b-22b, 24b** (1.6 μM). nt = nucleotides

Screening of fragments 12a-13a, 15a-22a and 24a for recognition by SNM1A at 5 mM in duplicate

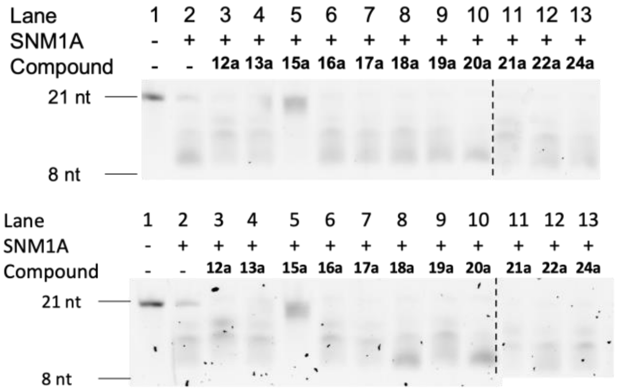

Figure S4. Evaluation of recognition of compounds **12a-13a, 15a-22a** and **24a** by SNM1A. Digestion of fluorescent substrate **11** (0.8 pmol) after incubation with SNM1A (50 fmol) for 60 min at 37 °C following 5 min preincubation with **12a-13a, 15a-22a** and **24a** (5 mM). nt = nucleotides.

4. NMR Spectra

^{1}H NMR (600 MHz, DMSO-*d6*) and ^{13}C (135 MHz, DMSO-*d6*) of **4**

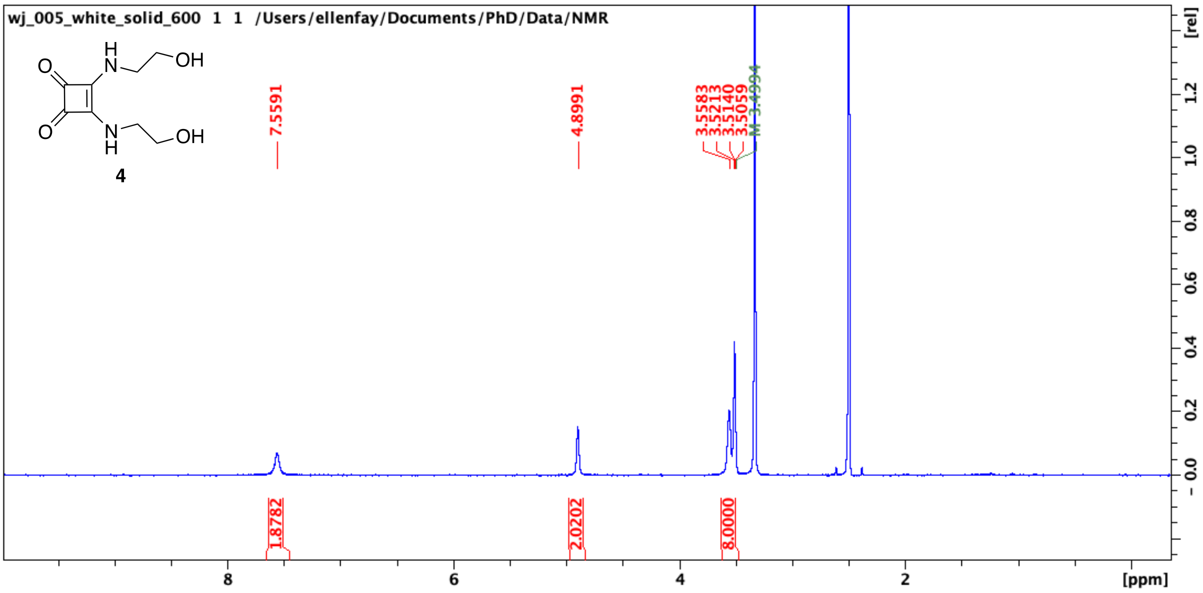

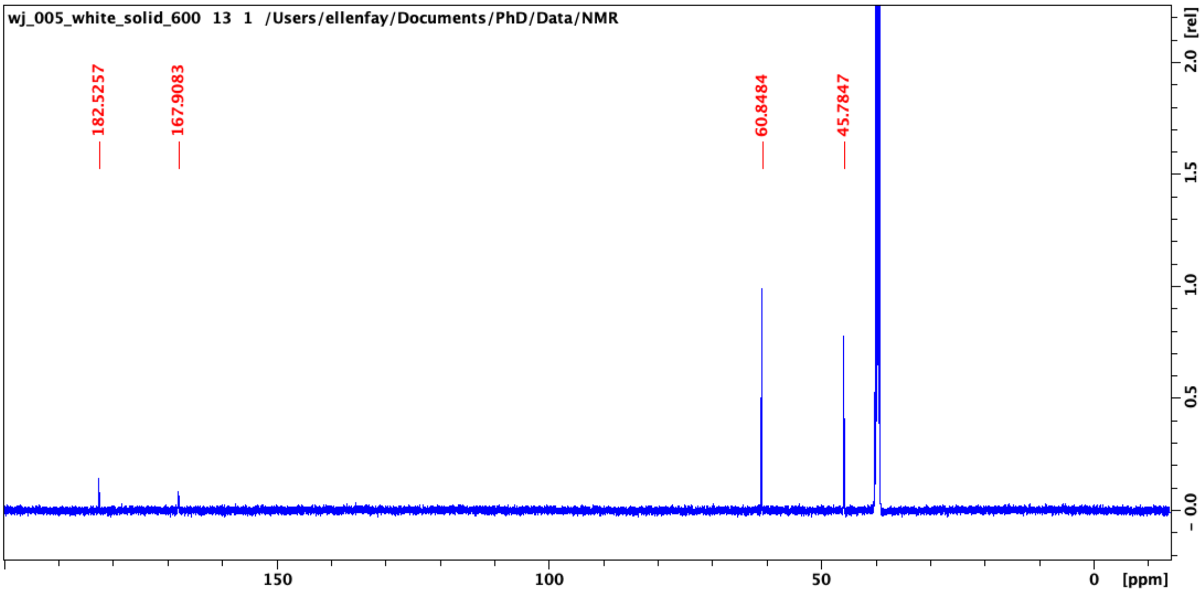

^{1}H NMR (400 MHz, DMSO-*d6*) and ^{13}C (135 MHz, DMSO-*d6*) of **5**

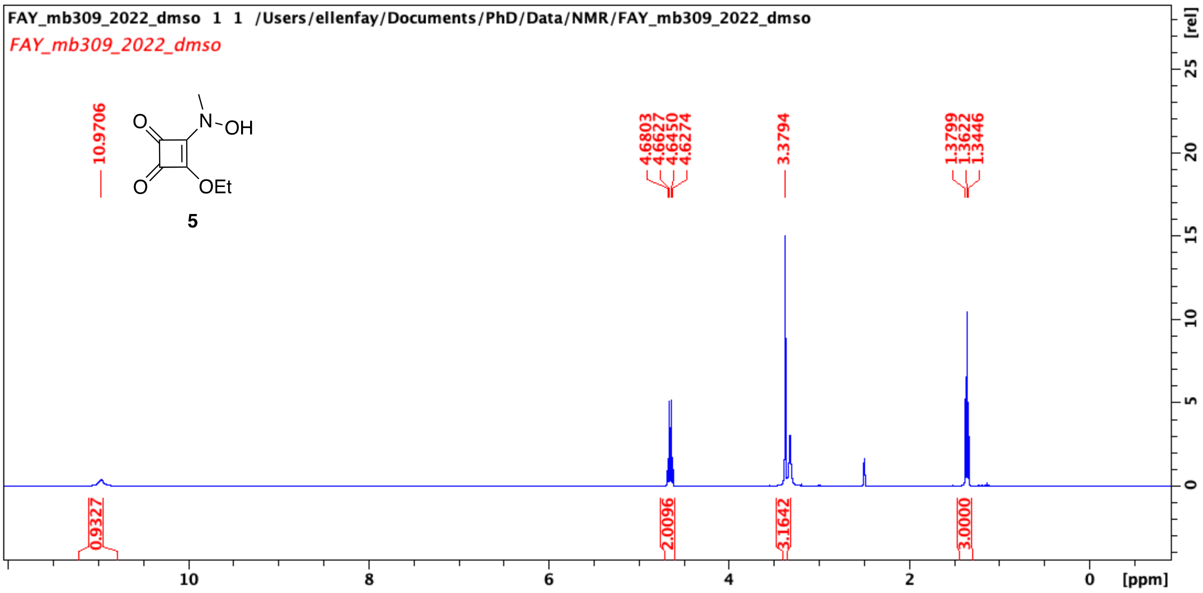

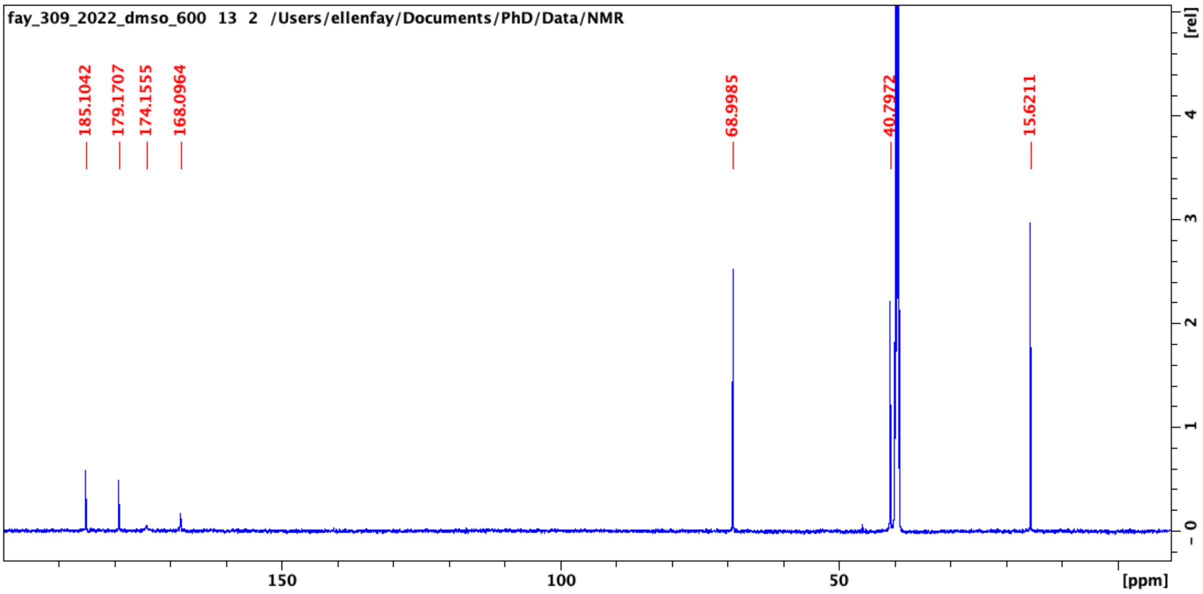

^{1}H NMR (400 MHz, DMSO-*d6*) and ^{13}C (101 MHz, DMSO-*d6*) of **7**

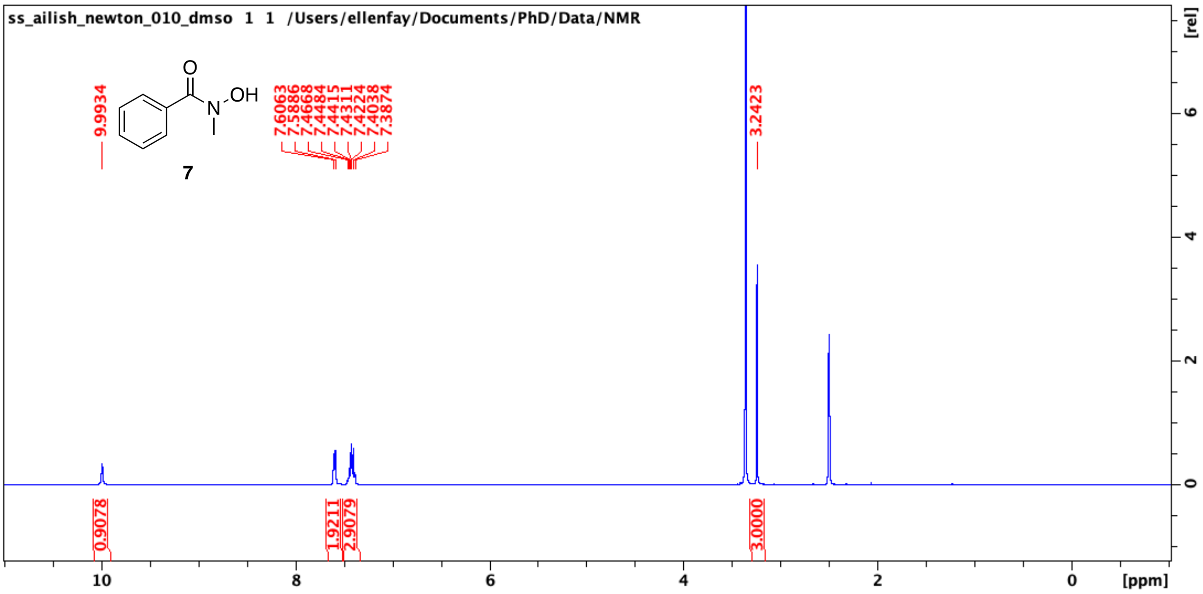

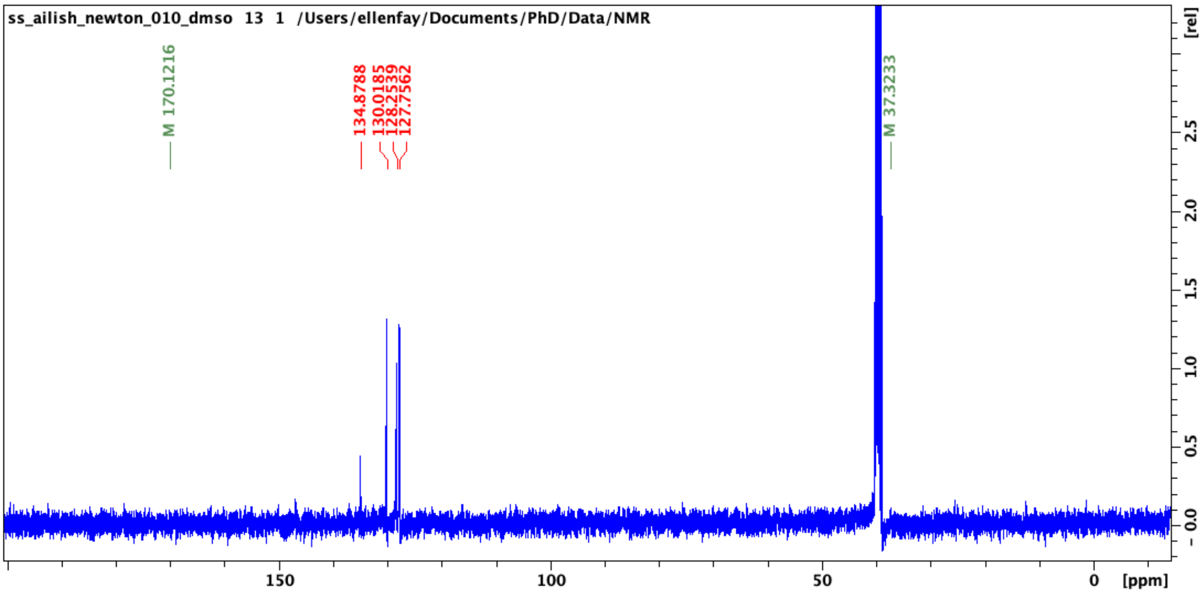

^{1}H NMR (400 MHz, CD_3OD) and ^{13}C (135 MHz, acetone-*d6*) of **8**

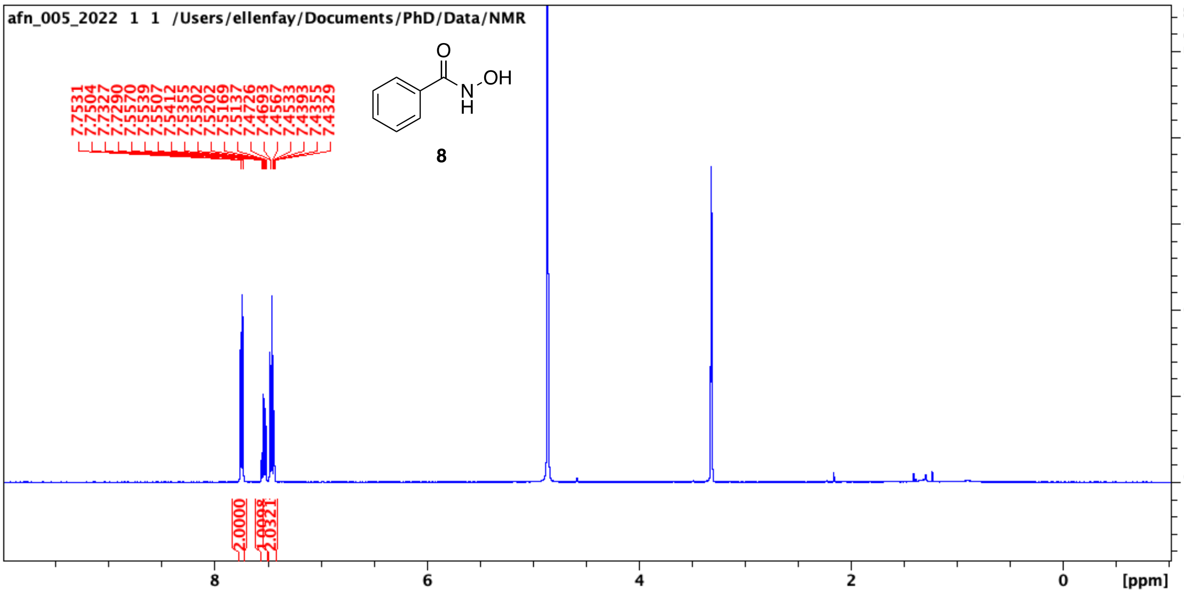

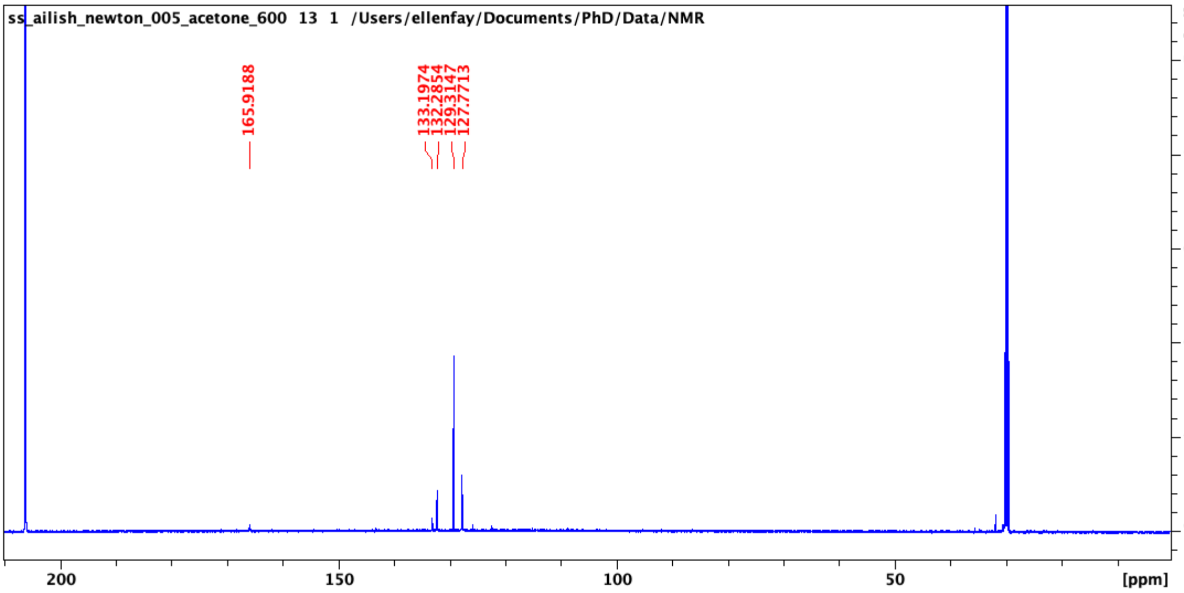

^{1}H NMR (400 MHz, $CDCl_3$) and ^{13}C (101 MHz, $CDCl_3$) of **13a**

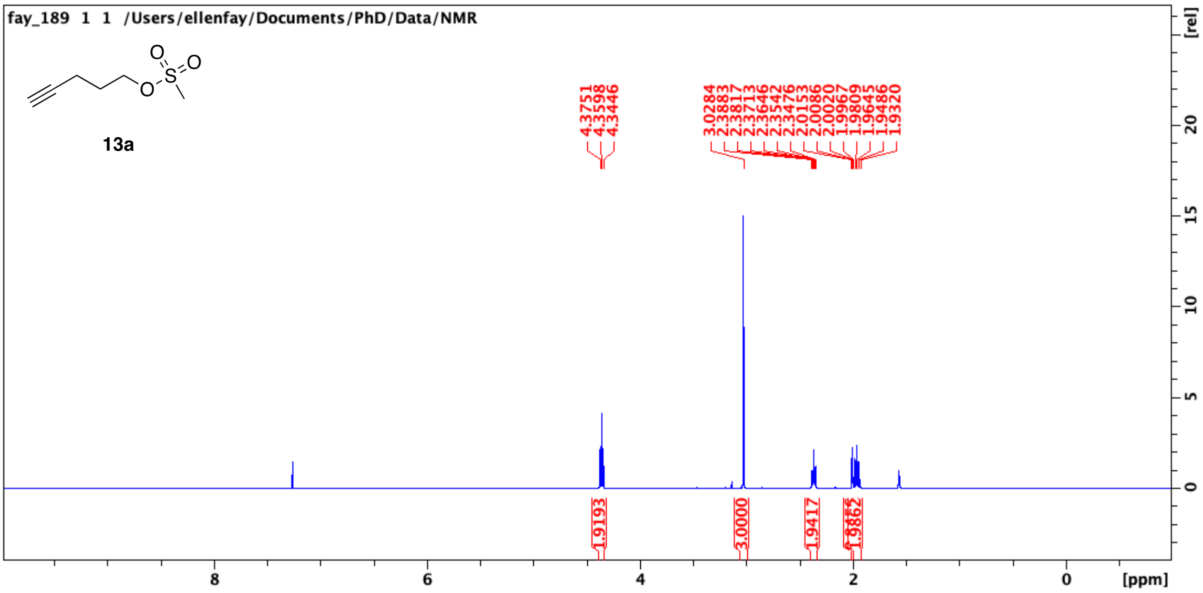

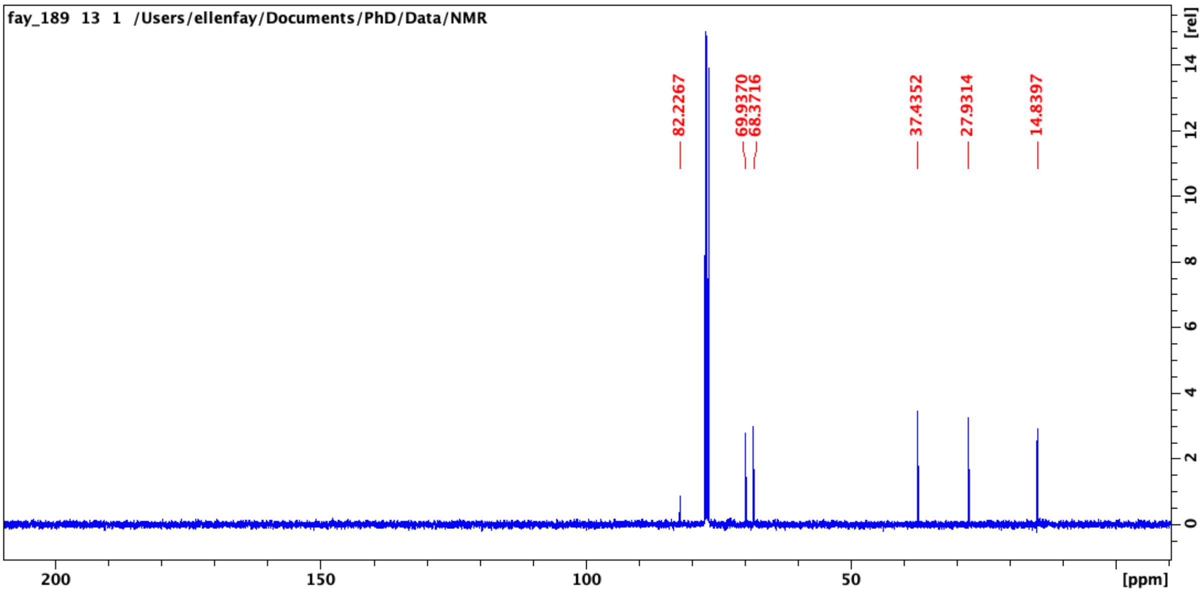

^{1}H NMR (400 MHz, $CDCl_3$) and ^{13}C (101 MHz, $CDCl_3$) of **14**

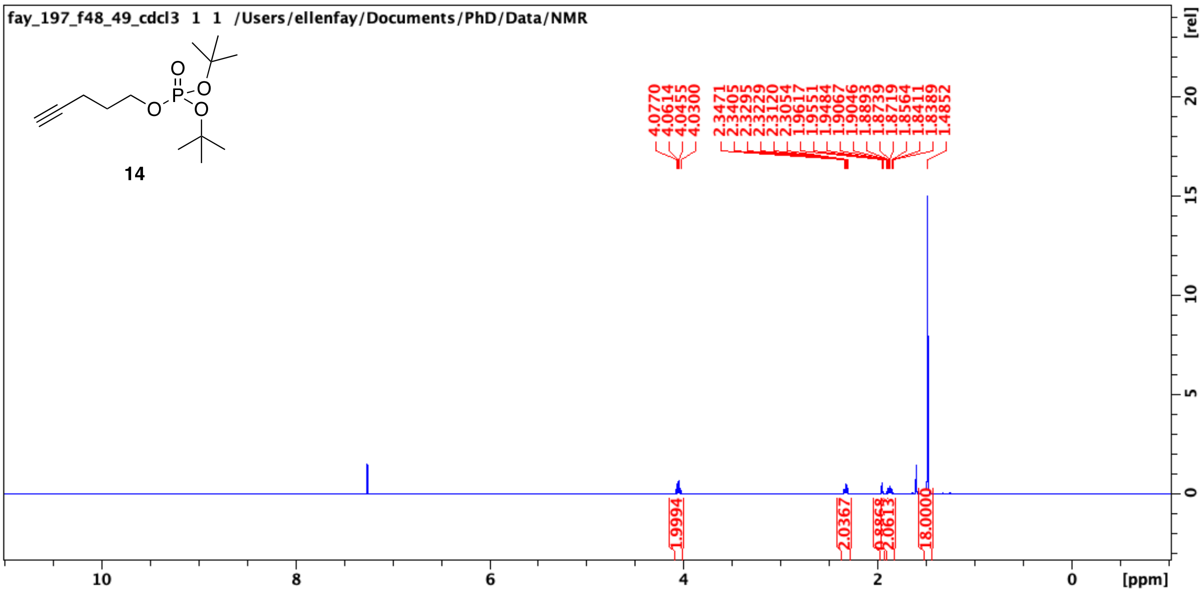

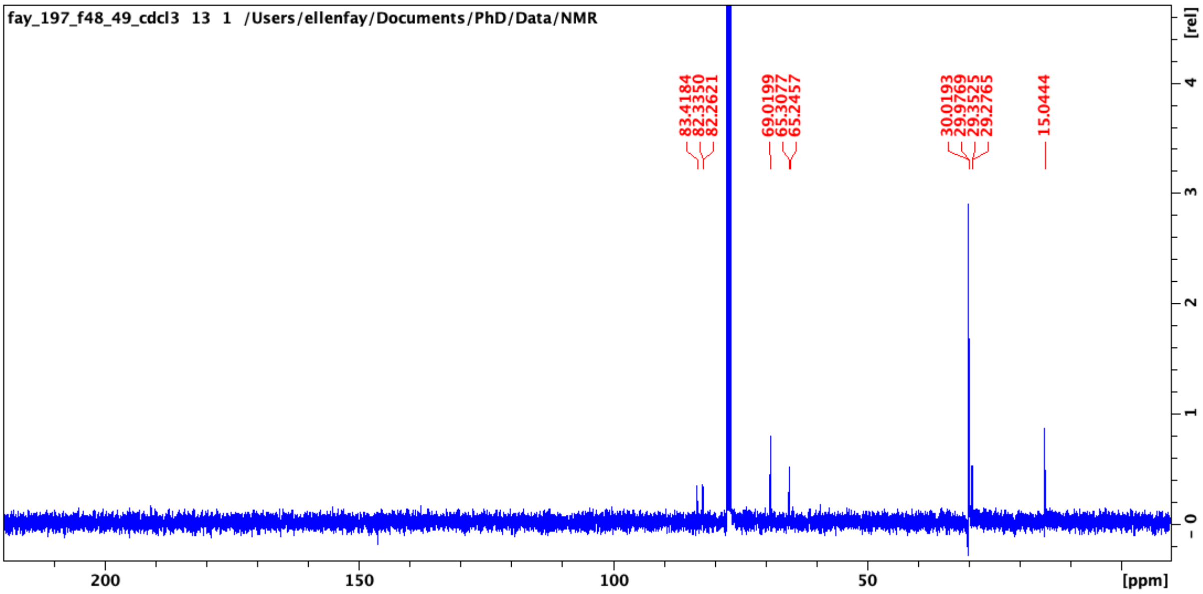

^{31}P NMR (162 MHz, $CDCl_3$) of **14**

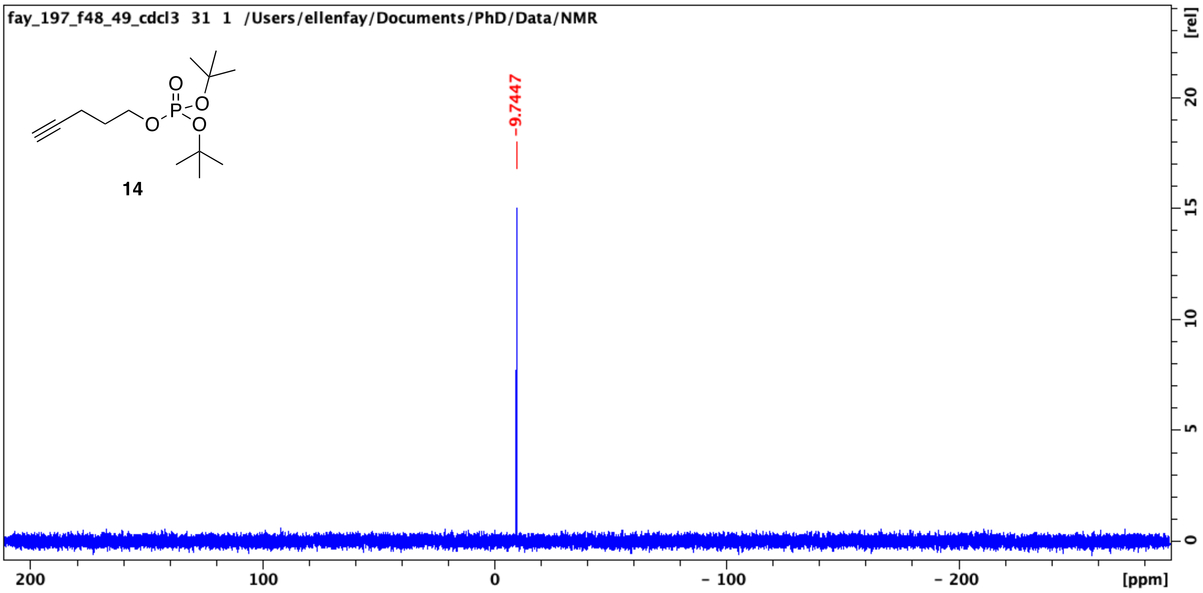

^{1}H NMR (400 MHz, CD_3OD) and ^{13}C (101 MHz, CD_3OD) of **15a**

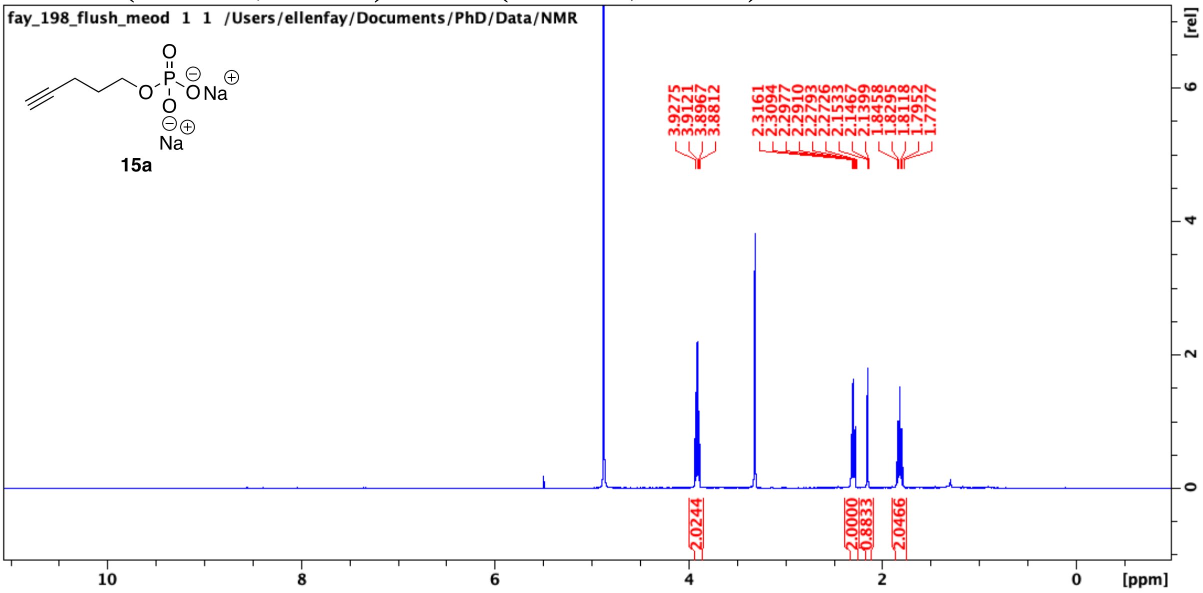

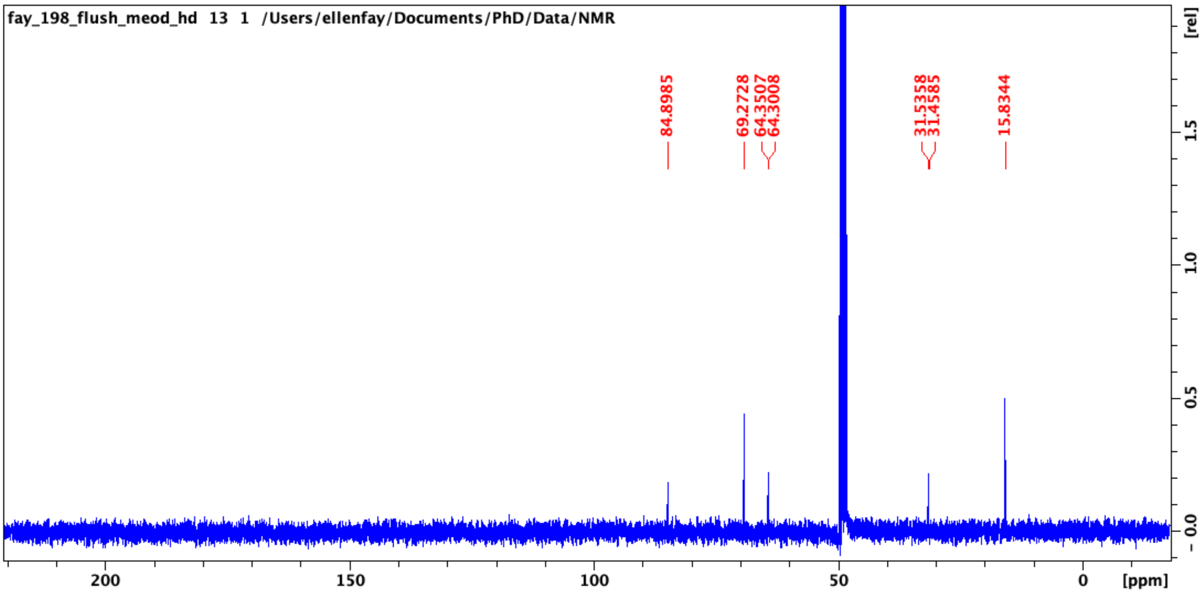

^{31}P NMR (162 MHz, $CDCl_3$) of **15a**

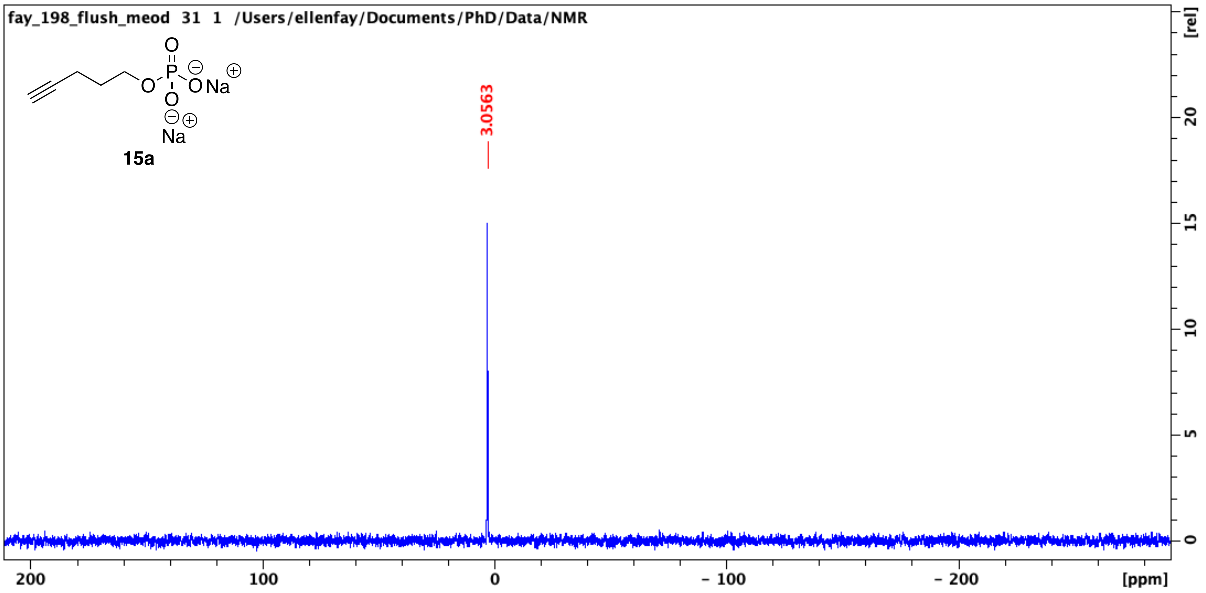

^{1}H NMR (400 MHz, $CDCl_3$) and ^{13}C (101 MHz, $CDCl_3$) of **17a**

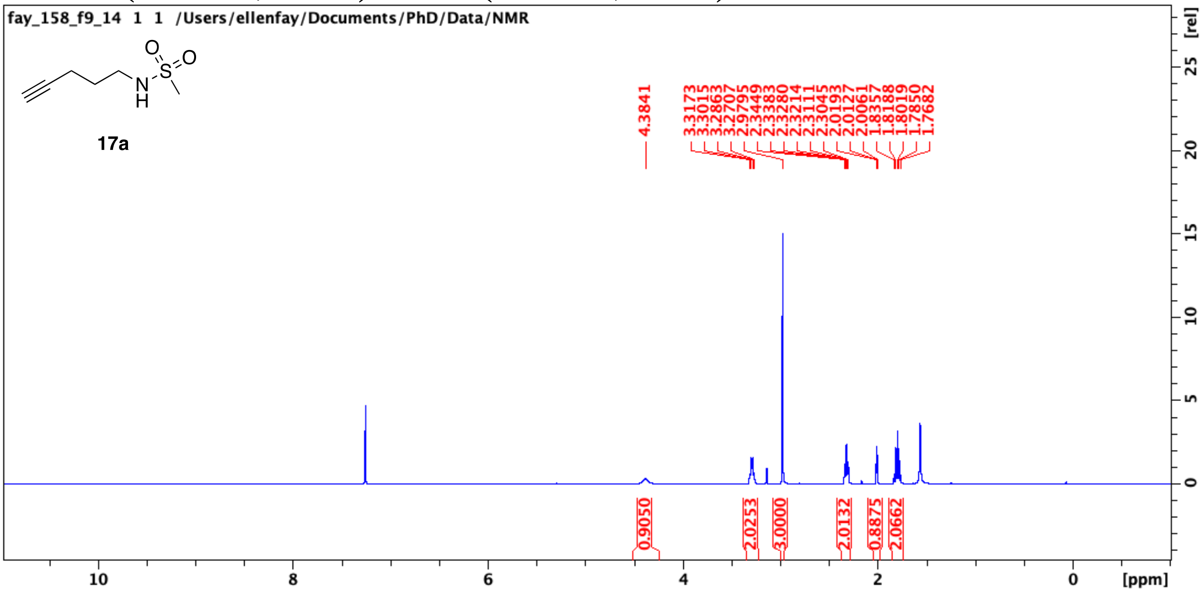

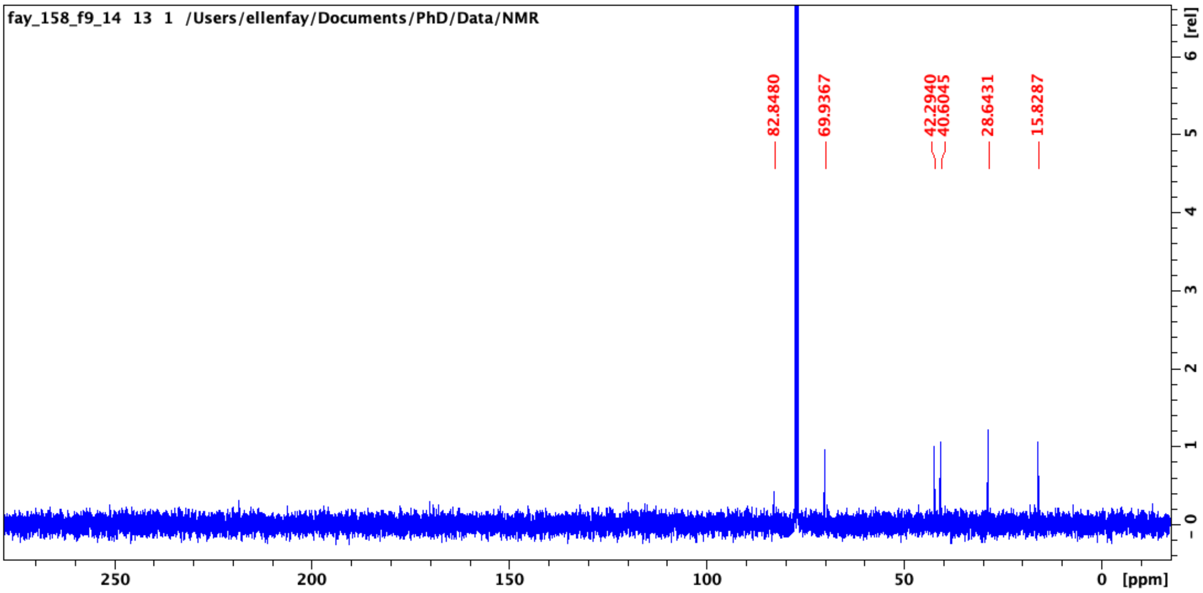

^{1}H NMR (400 MHz, $CDCl_3$) and ^{13}C (135 MHz, $CDCl_3$) of **18a**

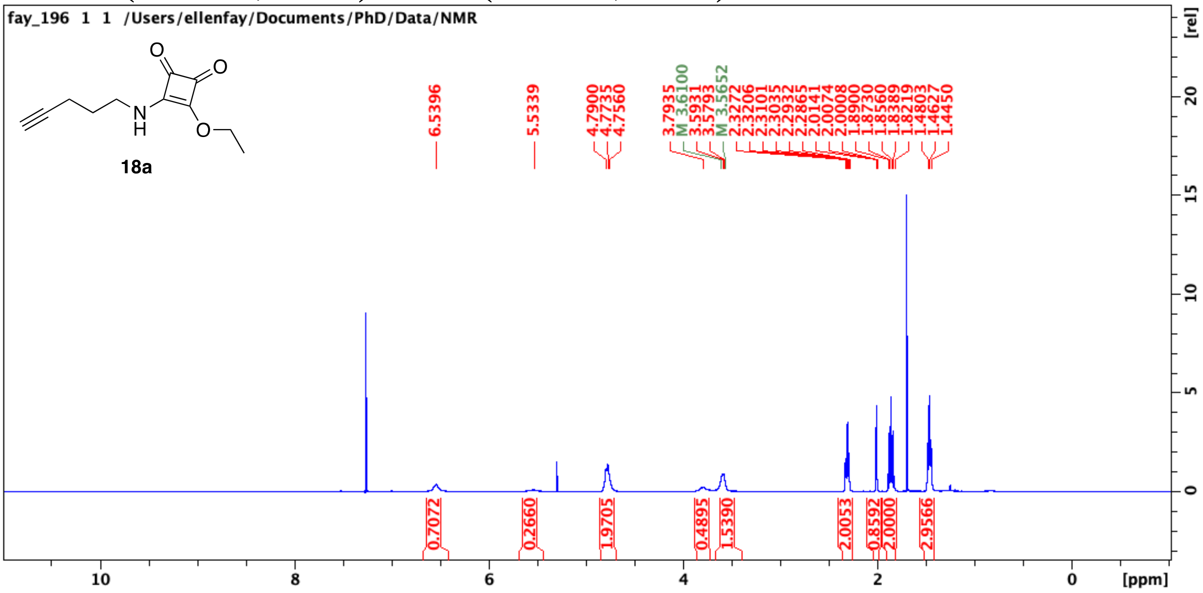

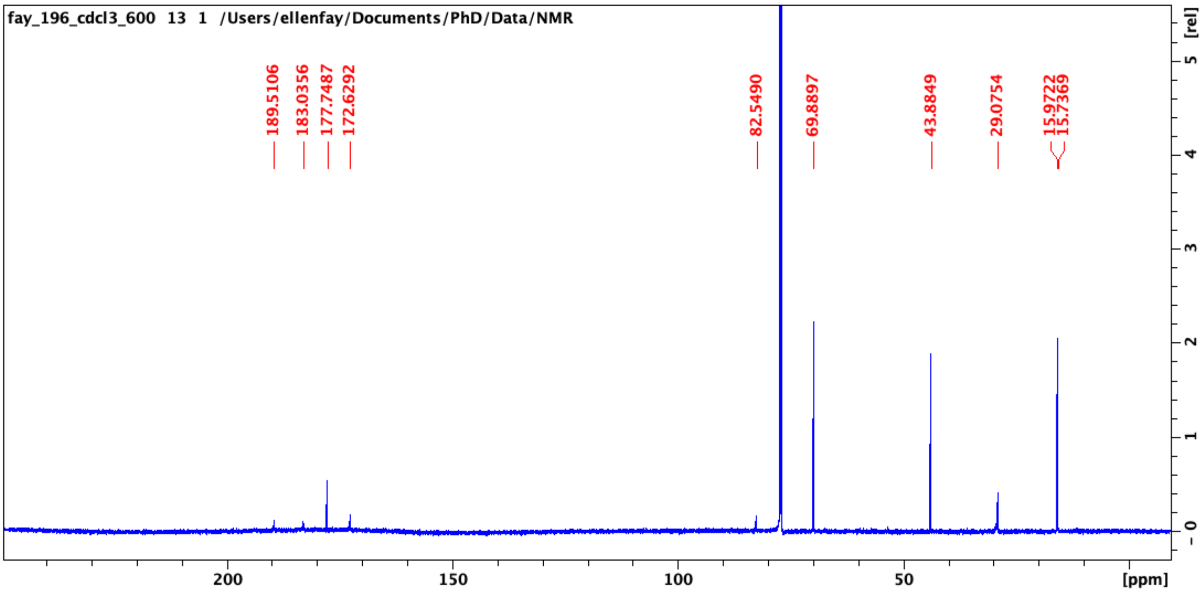

^{1}H NMR (400 MHz, DMSO-*d6*) and ^{13}C (135 MHz, DMSO-*d6*) of **19a**

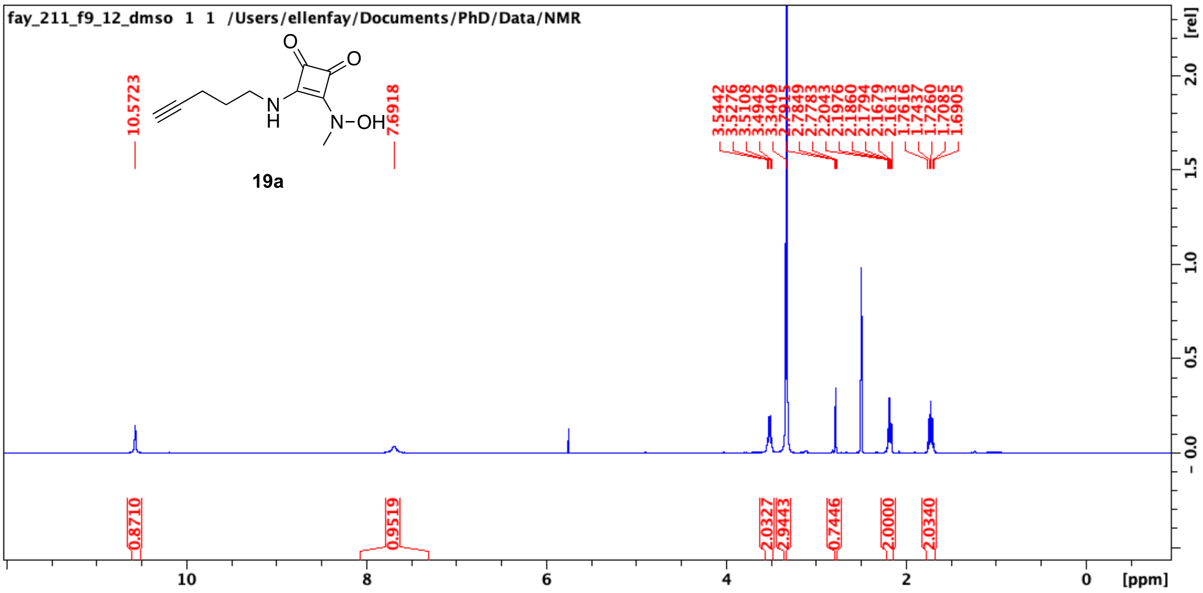

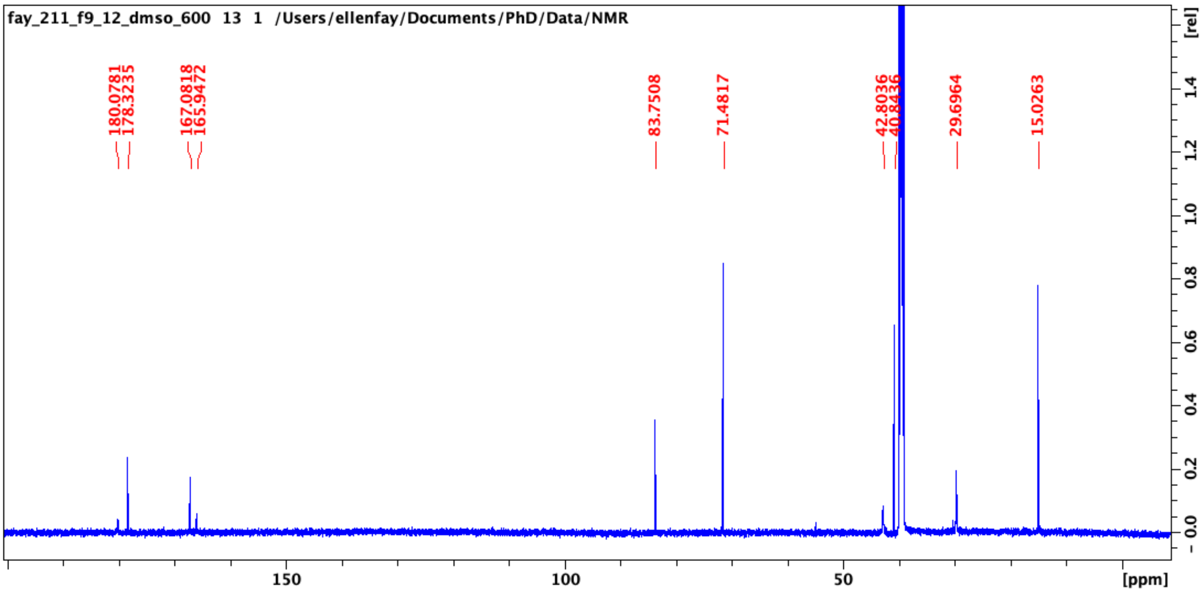

^{1}H NMR (600 MHz, DMSO-*d6*) and ^{13}C (135 MHz, DMSO-*d6*) of **20a**

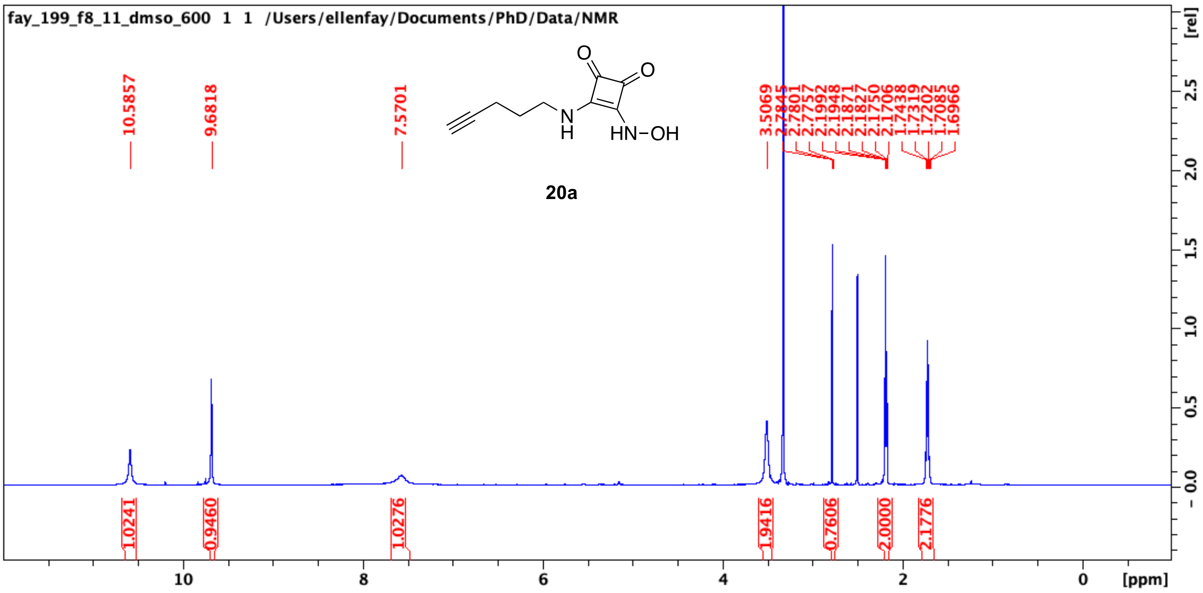

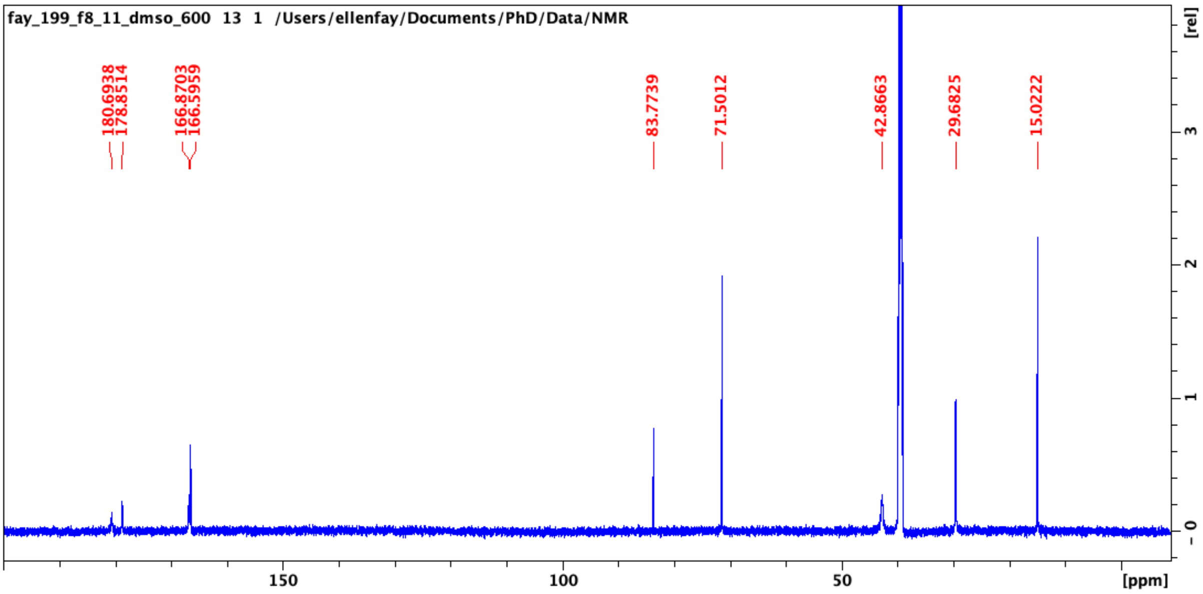

^{1}H NMR (400 MHz, $CDCl_3$) and ^{13}C (101 MHz, $CDCl_3$) of **22a**

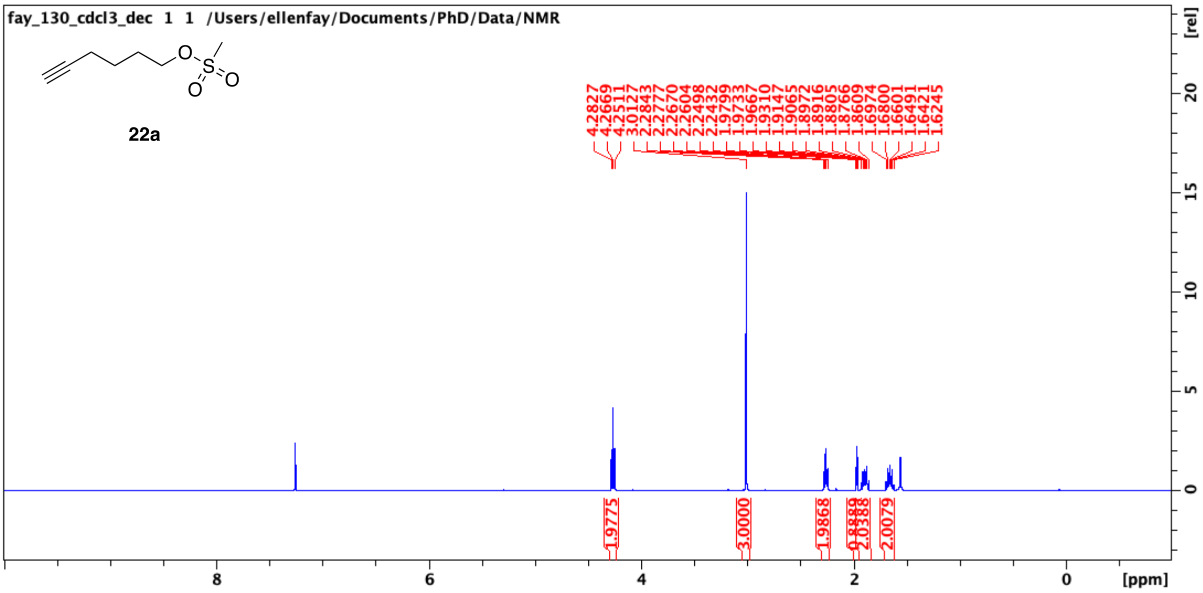

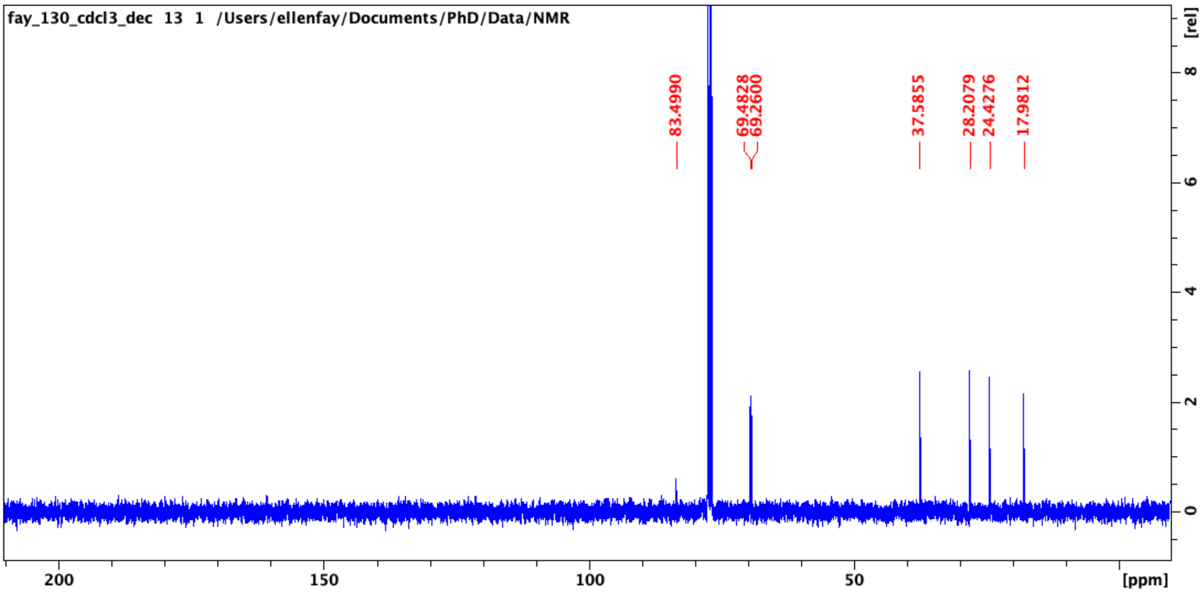

^{1}H NMR (400 MHz, $CDCl_3$) and ^{13}C (101 MHz, $CDCl_3$) of **24a**

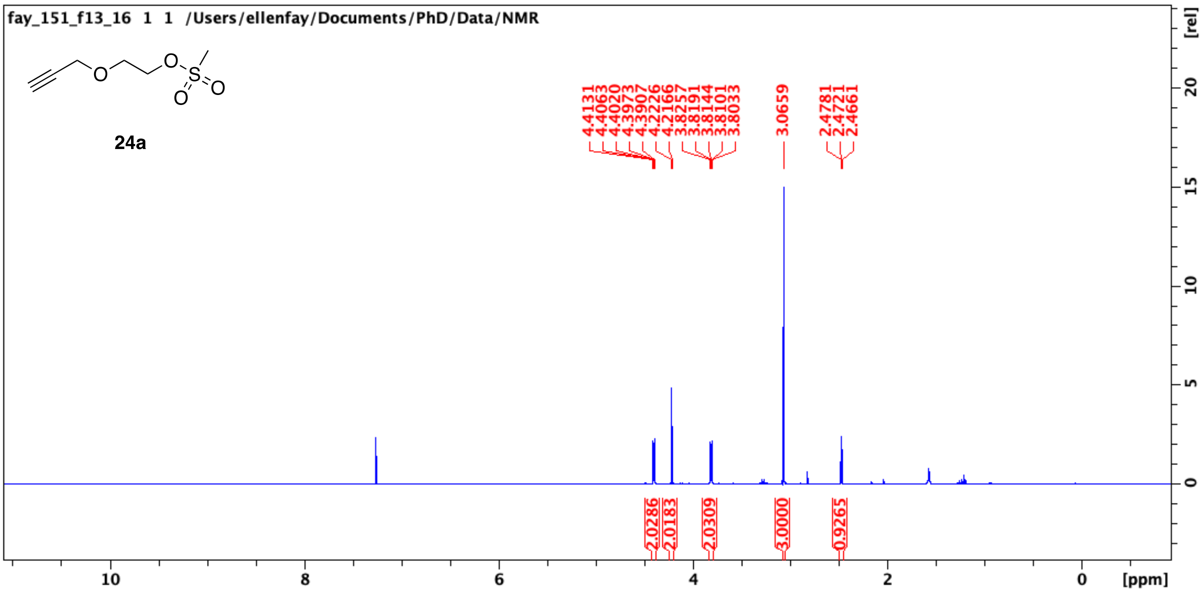

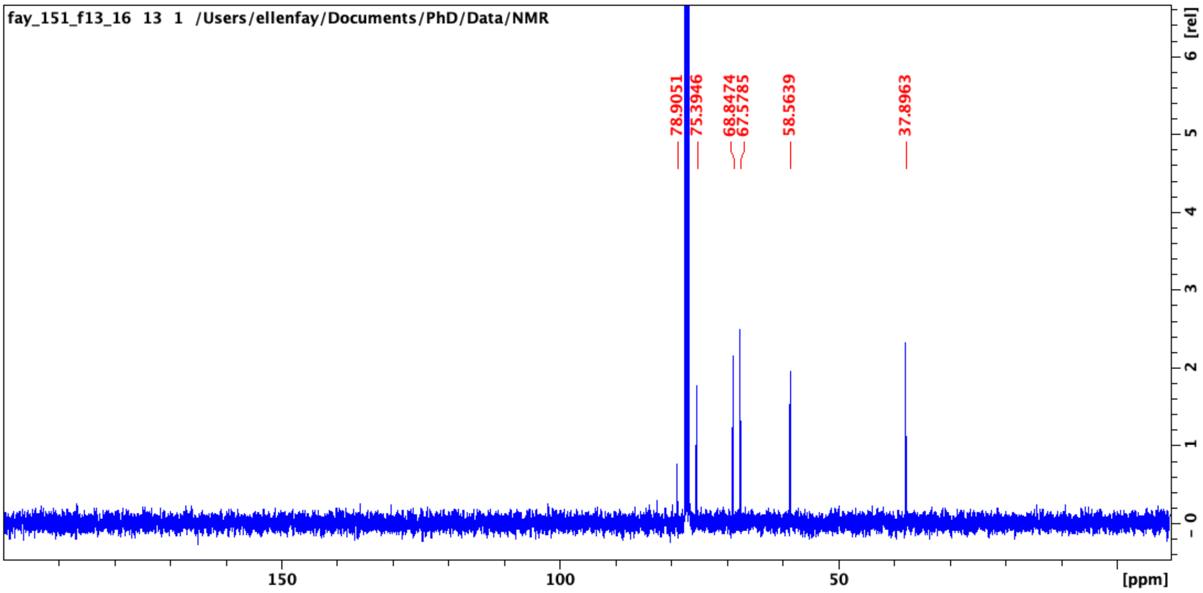

5. Mass spectra of modified oligonucleotides

Oligonucleotide	Modification	*m/z* Calc. [M+H]$^{+}$	Found
**12b**	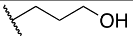	6227	6227
**13b**	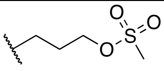	6305	6305
**15b**	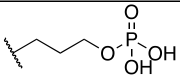	6307	6306
**16b**	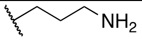	6226	6225
**17b**	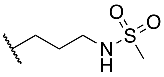	6304	6303
**18b**	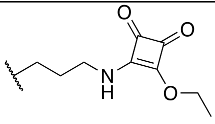	6350	6350
**19b**	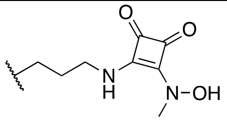	6351	6350*
**20b**	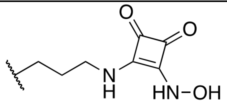	6337	6341*
**21b**	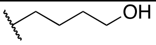	6241	6240
**22b**	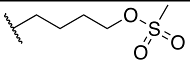	6319	6321**
**24b**	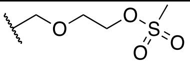	6321	6320

Table S1: Mass spectrometry data of the modified oligonucleotides **12b-13b, 15b-22b** and **24b** of the sequence 5'-(modifiedT) AG CAG TCA GTC AGT CAT GC-3'. *Main peak in mass spectra of *N*-hydroxysquaramides corresponds to 5'-amino oligonucleotide **16b** caused by fragmentation of the squaramide moieties. The intact mass is also seen in the mass spectra. **Main peak in mass spectrum of mesylate **22b** corresponds to a fragmentation product formed by elimination of the mesylate. The intact ion is also seen in the mass spectrum.

Oligonucleotides were used as a solution in H_2O (30-60 μM). A solution of 30 mg/mL 3-hydroxypicolinic acid (Sigma Aldrich) was freshly prepared in H_2O and used as a matrix. Diammonium citrate (30 mg/mL in H_2O) and diammonium tartrate (7.5 mg/mL in H_2O) were used as additives. A mixture of 1:1:1 oligonucleotide:matrix:additive was prepared to give a final oligonucleotide concentration of 10-20 μM. 1 μL of this mixture was spotted on the target plate using the dried droplet method. Positive and negative ion MALDI TOF spectra were obtained in linear mode on a Bruker 'autoflex maX' MALDI TOF/TOF instrument.

Alcohol 12b

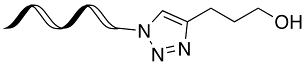

12b

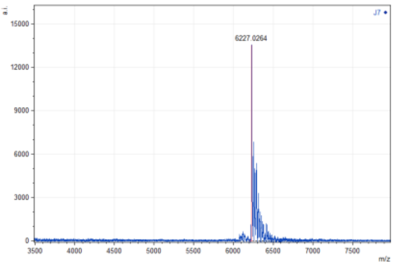

Mesylate 13b

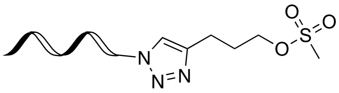

13b

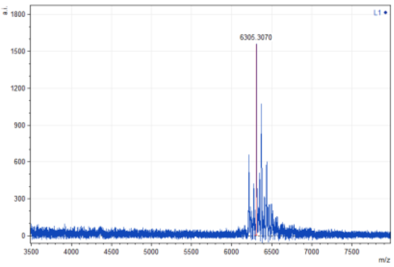

Phosphate 15b

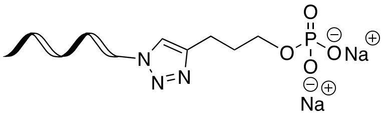

15b

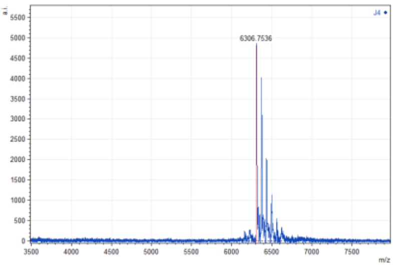

Amine 16b

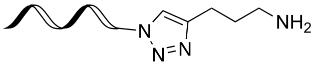

16b

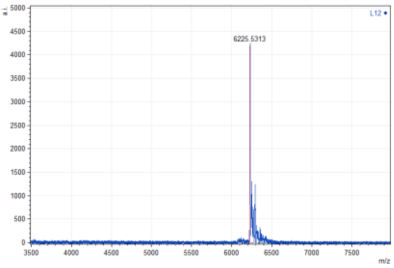

Sulfonamide 17b

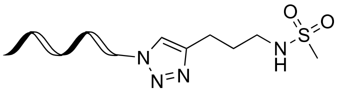

17b

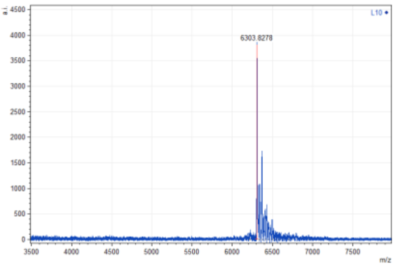

Squarylmonoamide 18b

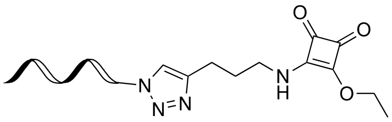

18b

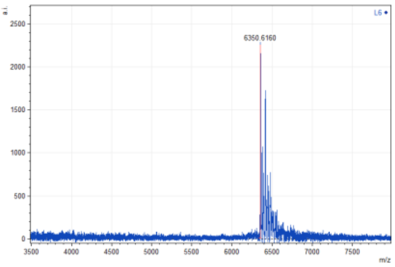

N-Methyl-*N*-hydroxyamino-squaryl diamide 19b

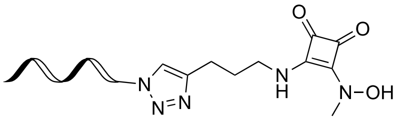

19b
m/z calc. 6350

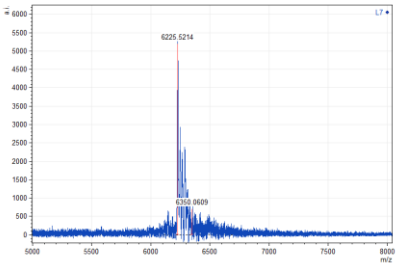

Fragment found:

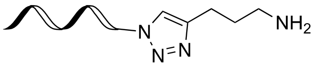

16b
m/z calc., 6225

N-Hydroxyamino-squaryl diamide 20b

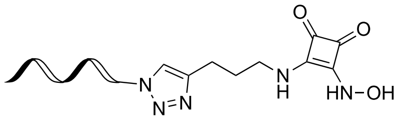

20b
m/z calc. 6337 $[M+H]^+$

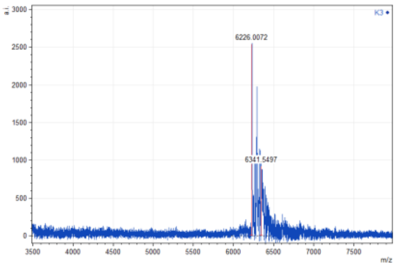

Fragment found:

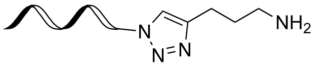

16b
m/z calc., 6226, $[M+H]^+$

Alcohol 21b

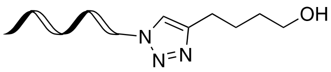

21b

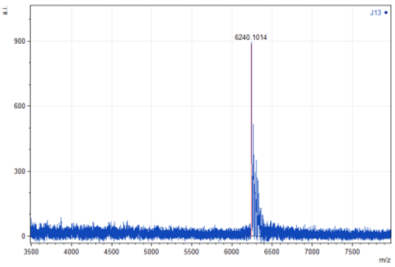

Mesylate 22b

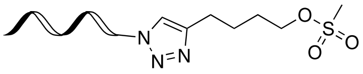

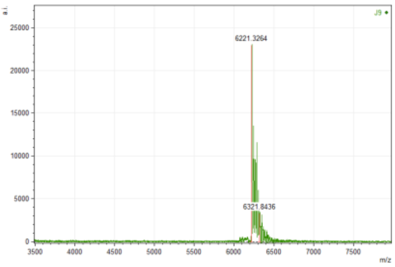

22b
m/z calc. 6319, [M+H]$^+$

Fragment found:

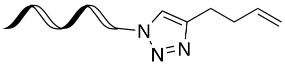

m/z calc. 6222

Mesylate 24b

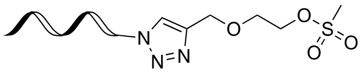

24b

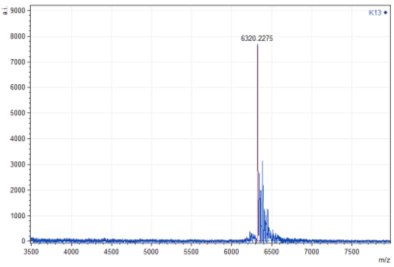

6. References

[1] G. Maahs, P. Hegenberg, *Angew. Chem.* **1966**, *78*, 927-931.

[2] G. X. Ortiz Jr, B. N. Hemric, Q. Wang, *Org. Lett.* **2017**, *19*, 1314-1317.

[3] R. Mocci, L. D. Luca, F. Delogu, A. Porcheddu, *Adv. Synth. Catal.* **2016**, *358*, 3135-3144.

[4] I. Delso, J. Valero-Gonzalez, F. Gomollón-Bel, J. Castro-López, W. Fang, I. Navratilova, D. M. van Aalten, T. Tejero, P. Merino, R. Hurtado-Guerrero, *ChemMedChem* **2018**, *13*, 128-132.

[5] Y. Yamamoto, Y. Nakanishi, K.-i. Yamada, K. Tomioka, *Tetrahedron* **2018**, *74*, 5309-5318.

[6] A. Vuorinen, I. V. Wilkinson, M. Chatzopoulou, B. Edwards, S. E. Squire, R. J. Fairclough, N. A. Bazan, J. A. Milner, D. Conole, J. R. Donald, *European Journal of Medicinal Chemistry* **2021**, *220*, 113431.

[7] E. M. Dürr, W. Doherty, S. Y. Lee, A. H. El-Sagheer, A. Shivalingam, P. J. McHugh, T. Brown, J. F. McGouran, *ChemistrySelect* **2018**, *3*, 12824-12829.
